# Telomere attrition predicts reduced survival in a wild social bird, but short telomeres do not

**DOI:** 10.1111/mec.15181

**Published:** 2019-08-07

**Authors:** Emma M. Wood, Andrew J. Young

**Affiliations:** ^1^ Centre for Ecology & Conservation University of Exeter Penryn UK

**Keywords:** life history, mortality, somatic maintenance, telomere dynamics

## Abstract

Attempts to understand the causes of variation in senescence trajectories would benefit greatly from biomarkers that reflect the progressive declines in somatic integrity (SI) that lead to senescence. While telomere length has attracted considerable interest in this regard, sources of variation in telomere length potentially unrelated to declines in SI could, in some contexts, leave telomere attrition rates a more effective biomarker than telomere length alone. Here, we investigate whether telomere length and telomere attrition rates predict the survival of wild white‐browed sparrow‐weaver nestlings (*Plocepasser mahali*). Our analyses of telomere length reveal counterintuitive patterns: telomere length soon after hatching negatively predicted nestling survival to fledging, a pattern that appears to be driven by differentially high in‐nest predation of broods with longer telomeres. Telomere length did not predict survival outside this period: neither hatchling telomere length nor telomere length in the mid‐nestling period predicted survival from fledging to adulthood. Our analyses using within‐individual telomere attrition rates, by contrast, revealed the expected relationships: nestlings that experienced a higher rate of telomere attrition were less likely to survive to adulthood, regardless of their initial telomere length and independent of effects of body mass. Our findings support the growing use of telomeric traits as biomarkers of SI, but lend strength to the view that longitudinal assessments of within‐individual telomere attrition since early life may be a more effective biomarker in some contexts than telomere length alone.

## 1 INTRODUCTION

Senescence, the progressive late‐life decline in reproductive success or survival now widely documented across taxa, is thought to arise from age‐related declines in Somatic Integrity (SI; the extent to which somatic tissues are free from biomolecular errors and damage; Kirkwood, 1977; Kirkwood & Holliday, 1979). Attempts to understand the proximate and ultimate causes of variation in senescence trajectories would therefore benefit greatly from the ability to estimate SI and its rate of decline over time (Kirkwood, 2005). Diverse lines of evidence highlight the possibility that telomere length could provide a useful biomarker of SI (Boonekamp, Simons, Hemerik, & Verhulst, 2013; Young, 2018). For example, telomere length can positively predict survival in humans (Cawthon, Smith, O'Brien, Sivatchenko, & Kerber, 2003; Kimura et al., 2008) and natural animal populations (see Appendix S1 for a collation of studies of both survival and lifespan) both in early life and adulthood, findings consistent with it serving as a biomarker of SI, given the expectation that deficits in SI accumulate over the life course (Kirkwood, 1977) and could reduce survival at any life stage by compromising resilience to environmental hazards. However, there is growing recognition that single measures of telomere length may be of limited utility in this regard as various processes have the potential to generate interindividual differences in telomere length estimates independent of variation in SI. Indeed, positive associations between telomere length and survival are far from universal (S1), and a recent meta‐analysis concluded that while a significant positive association does exist across studies (as one would predict if telomere length does reflect SI), the overall effect size is small and exhibits significant heterogeneity across studies (Wilbourn et al., 2018). Complementing measures of telomere length with longitudinal assessments of telomere attrition experienced since early life (e.g., see Boonekamp, Mulder, Salomons, Dijkstra, & Verhulst, 2014) could improve attempts to characterize SI at any point in the lifespan, by overcoming limitations arising from interindividual variation in telomere length estimates unrelated to SI.

Telomeres are ribonucleoprotein structures that cap the ends of linear chromosomes, buffering coding DNA from the gradual erosion that occurs during cell replication (de Lange, 2009; Olovnikov, 1973). In the absence of telomere repair mechanisms, telomeres get shorter with each cell division, and accumulation of critically short telomeres within a cell can trigger apoptosis and cellular senescence (Blackburn, 2000; Hemann, Strong, Hao, & Greider, 2001; a cell fate now causally implicated in organismal senescence, Baar et al., 2017; Baker et al., 2016). At least three processes have the potential to leave telomere length correlated with, and hence a useful biomarker of, SI. First, individuals with shorter telomeres might be expected to have reduced SI if the accumulation of critically short telomeres is a major proximate cause of cellular senescence in vivo. However, whether this is the case remains unclear: while cellular senescence *in vivo* may commonly be triggered by telomeric mechanisms (Herbig, Ferreira, Condel, Carey, & Sedivy, 2006; Hewitt et al., 2012), recent findings suggest that such telomeric triggering may occur via length‐independent mechanisms (Hewitt et al., 2012; Young, 2018). Second, processes that accelerate telomere shortening may also hasten the accumulation of damage to other biomolecules in somatic tissues. For example, oxidative damage can accelerate telomere shortening directly by causing breaks in telomeric DNA, the repair of which may be suppressed to some extent (Jia, Her, & Chai, 2015; Kawanishi & Oikawa, 2004; Petersen, Saretzki, & Zglinicki, 1998; Von Zglinicki, 2002). Telomere shortening could thereby reflect the correlated accumulation of oxidative damage to other biomolecules in somatic tissues. Finally, any cause of biomolecular damage that also increases the incidence of cell death has the potential to accelerate telomere shortening, if such cellular losses trigger compensatory increases in the rate of stem cell division and any associated telomere attrition (Young, 2018).

While telomere length per se might thereby constitute a useful biomarker of SI, multiple sources of interindividual variation in telomere length estimates potentially unrelated to within‐individual declines in SI highlight a need for caution. For example, evidence of apparent genetic and epigenetic variation in telomere length has highlighted the possibility of variation in the notional “initial” telomere length set during development (e.g., at conception; Dugdale & Richardson, 2018; Eisenberg & Kuzawa, 2018; Olsson et al., 2011). If such mechanisms yield interindividual variation in “initial” telomere length *independent* of SI (a matter that remains poorly understood, e.g., see Dugdale & Richardson, 2018), this would be expected to weaken any later‐life association between telomere length per se and the within‐individual declines in SI that telomere attrition might nevertheless effectively track (Boonekamp et al., 2014). Furthermore, the extent of interstitial telomeric sequences (i.e., those found *within* chromosomes) could contribute significantly to telomere length estimates obtained via quantitative PCR approaches, thereby obscuring the true relationship between telomere length and SI (Criscuolo et al., 2009; Delany, Daniels, Swanberg, & Taylor, 2003; Foote, Vleck, & Vleck, 2013). The utility of telomere length per se as a biomarker of SI may therefore depend upon the extent to which such factors contribute to individual variation in telomere length, and this could well vary among species and across the life course.

A potential solution to this problem would be to complement telomere length measures with longitudinal assessments of telomere attrition since early life. Indeed, while few studies of natural populations have investigated the prognostic value of both telomeric traits together (see Appendix S1), one such study did find that offspring survival was predicted by telomere attrition rate in early life but not telomere length (Boonekamp et al., 2014). Advances in our understanding of the utility of telomeric traits as biomarkers of SI, and the mechanistic reasons for their predictive utility, could therefore be well served by further investigations of the extent to which both telomere length and telomere attrition from early life predict performance in the wild.

Here, we use data from a natural population of white‐browed sparrow‐weavers (*Plocepasser mahali*) to investigate whether telomere length and attrition rate during the nestling stage predict early life survival. First, we investigate whether nestling telomere length at 4 and 12 days of age predicts their downstream survival to the start of the following breeding season (i.e., adulthood). Second, we investigate whether rates of telomere attrition between 4 and 12 days of age predict survival to the following breeding season, while controlling for telomere length at 4 days of age. In all analyses, we control for effects of body mass. Viewing telomere attrition in early life as a biomarker of within‐individual declines in SI, we predict that nestlings that show higher rates of telomere attrition will have reduced downstream survival even after controlling for variation in body mass. Whether such a relationship between telomere attrition and survival also gives rise to a positive association between telomere length and downstream survival will depend in part upon the extent to which variation in telomere length at the focal time points arises from mechanisms other than within‐individual declines in SI. Indeed, if variation in both SI and telomere length principally arises from developmental differences at the egg stage, telomere length at 4 days of age could be a stronger predictor of downstream survival than subsequent telomere attrition.

## 2 METHODS

### 2.1 Study species and field methods

This work was conducted as part of a long‐term field study of 38 social groups of white‐browed sparrow‐weavers (*P. mahali*) in the semi‐arid Kalahari Desert at Tswalu Kalahari Reserve, South Africa (27°16′S, 22°25′E). White‐browed sparrow‐weavers are year‐round territorial cooperatively breeding birds that live in social groups of 2–14 individuals (Harrison, York, Cram, Hares, & Young, 2013) and can live for in excess of 12 years (pers. obs. E. M. Wood & A. J. Young). Reproduction within the group is monopolized by a single dominant pair (Harrison, York, Cram, Hares, et al., 2013; Harrison, York, Cram, & Young, 2013) while subordinate group members contribute to a range of cooperative activities including feeding nestlings (Cram, Blount, & Young, 2015; Walker, York, & Young, 2016). Throughout the breeding season (October to April inclusive), all nests were closely monitored to attain lay and hatch dates for all eggs. Nestlings were assigned unique markings by trimming the downy feathers on their heads, allowing them to be distinguished until they were large enough to receive a uniquely numbered metal leg ring at 12 days of age (safring licence 1444). Broods were then checked on the days on which the first‐hatched nestling in the brood would have been 4, 8, 12 and 16 days old. Henceforth, the age classes of nestlings are referred to according to the age that the first‐hatched chick in their brood would have been on that day (i.e., “day 4” nestlings are those sampled when the first‐hatched nestling in their brood was 4 days old). As brood sizes rarely exceeded two nestlings, which near‐invariably hatched on the same or successive days, these age classes typically captured true nestling age to within a day (see below). The precise ages of individual focal nestlings at sampling were nevertheless controlled statistically in each of the analyses.

On days 4 and 12, a small blood sample (<25 μl) was collected from nestlings via brachial venepuncture using a 26G needle and nonheparinized capillary tube and stored in absolute ethanol at ambient temperature until extraction. Nestling body mass was also recorded to the nearest 0.01 g (Durascale 100; MyWeigh). To avoid the risk of prefledging the brood, nestlings were not disturbed again after day 16, but groups were monitored closely to establish whether nestlings survived to fledging (which typically occurs at 20–25 days of age; see below). The sexes of birds that survived to adulthood were determined by beak colour, which is sexually dimorphic in this subspecies (Leitner, Mundy, & Voigt, 2009), otherwise molecular sexing was used (Dawson, 2007; 87 nestlings, see Appendix S2.1). All protocols were approved by the University of Pretoria Ethics Committee and conform with the guidelines for the use of animals in research.

### 2.2 Survival assessments

Survival of nestlings was recorded during standard nest checks (described above). From 3 weeks after the first nestling hatched, groups were thoroughly checked for the presence of fledglings. Fledgling identity was confirmed upon capture at ~30 days of age, when they were fitted with colour rings for downstream monitoring. Nestlings were recorded as having not survived to fledging if they were never observed outside the nest. The survival of birds to the start of the following breeding season (i.e., adulthood; defined as 1st October, following the season in which they hatched) was determined during observations carried out at least every other week at each group throughout the breeding season (Harrison, York, Cram, Hares, et al., 2013). Fledglings very rarely disperse from their natal group prior to the start of the next breeding season (8 of 429 fledglings over 8 years), and dispersal in this species is unusually local in both sexes, occurring extensively within the bounds of our study site (Harrison, York, & Young, 2014). It is therefore unlikely that significant numbers of fledglings that were considered nonsurvivors instead dispersed beyond the bounds of our study population.

### 2.3 DNA extraction and telomere measurement by qPCR

We extracted DNA using Gentra PureGene Genomic DNA Purification Kits (Qiagen) and discarded samples with poor DNA integrity or purity (Appendix S2.2). We used quantitative PCR (qPCR) as described in Cawthon (2002; with modifications described below) to quantify telomere length relative to a nonvariable copy‐number control gene (termed RTL for brevity in the 0 and results tables, but “telomere length” for clarity in the results text and discussion). For the control gene, we used glyceraldehyde‐3‐phosphate‐dehydrogenase (GAPDH), using primers specific to *P. mahali* (GAPDH‐f 5′AAACCAGCCAAGTATGATGACAT–3′; GAPDH‐R 5′‐CCATCAGCAGCAGCCTTCA‐3′). Telomere primers were Tel1b (5′ CGGTTTGTTTGGGTTTGGGTTTGGGTTTGGGTTTGG GTT‐3′) and Tel2b (5′‐GGCTTGCCTTACCCTTACCCTTACCCTTACCCTTACCCT‐3′). Each 20 μl reaction contained a total of 5 ng DNA (for both GAPDH and telomere reactions) and 10 μl SYBR Green fluorescent dye with low ROX (Agilent Technologies), with primers at a concentration of 200 nM. DNA pooled from three individuals was used for the standard curve and as a between‐plate calibration sample. All samples and no template controls (on each plate) were run in triplicate on a Stratagene Mx3000 instrument. Thermal cycles for telomere reactions were 15 min at 95°C, followed by 40 cycles of 95°C for 15 s, 57°C for 30 s and 73°C for 30 s. Thermal cycles for GAPDH were the same, except the annealing temperature which was 60°C. LinRegPCR (Ruijter et al., 2009) was used to correct baseline fluorescence, determine the window of linearity for each amplicon and calculate individual well efficiencies. We calculated RTL following Pfaffl (2001). Intraplate coefficients of variation were 0.31% (*SD* = 0.16) for GAPDH and 0.83% (*SD* = 0.50) for telomere. Interplate co‐efficient of variation of RTL was 13.09% (*SD* = 8.15). See Appendix S2.3 for further details.

### 2.4 Statistical analyses

Statistical analyses were carried out in “r” (version 3.3.1). For all analyses, we adopted an information theoretic (IT) model selection approach, using Akaike information criterion correcting for small sample size (AICc) to compare models (Burnham & Anderson, 2002). Global models were constructed that included all variables of interest (continuous predictors were centred and scaled), and variance inflation factors (VIFs) were used to assess multicollinearity in each global model; all VIFs were below 5 (“car” package; Fox & Weisberg, 2011). As all our predictor variables have the potential for independent additive effects on the response variables of interest, the fits of all combinations of the predictor variables were compared and ranked based on AICc using the package mumin (Barton, 2016). Two‐way interactions and quadratic terms were only included if such relationships were considered plausible a priori (see below) and were accompanied in all relevant models by the corresponding first‐order terms. We retained all models within Δ6 AICc of the top model (referred to collectively as the top model set), as this allows confidence that the most parsimonious model is included, but removes models with only very weak support (Richards, 2005). In order to avoid retention of overly complex models, those that were more complex versions of better performing models were excluded (Richards, 2008; Richards, Whittingham, & Stephens, 2011); top model sets prior to implementation of the nesting rule are presented in the Appendix S3.2. We checked for overdispersion in all global models. In all cases, the point estimate of dispersion was below 1.5.

#### 2.4.1 Does telomere length predict survival?

We conducted two binomial glmms (glmer function in the package “lme4”, with the bobyqa optimiser; Bates, Mächler, Bolker, & Walker, 2015) to test whether RTL on days 4 and 12 of the nestling period predicted survival to the start of the following breeding season. We fitted the following terms as covariate predictors in order to control for their potential effects on survival: nestling body mass (see below), sex, age at sampling (see below), adult group size at hatching (number of group members over 6 months of age), two measures of rainfall (rainfall over the month prior to egg‐laying: “prelay rainfall”, and rainfall over the period from egg‐laying to day 30 of the nestling period: “postlay rainfall”), and the number of days between hatching and the next 1 October. This latter variable controls for effects of variation in the length of time that nestlings had to survive in order to be classed as having survived to the start of the following breeding season, as nestlings could hatch at any time during a given breeding season (though mechanisms other than simply having to survive for longer could also contribute to such an association). Social group, breeding season and brood identity were included as random factors. The analysis of the effects of day 4 RTL on survival utilized a sample size of 82 nestlings from 59 broods reared by 30 social groups over three breeding seasons, while that for day 12 RTL utilized a larger sample size of 146 nestlings from 100 broods reared by 36 social groups over five breeding seasons.

The hatch dates for all nestlings were known to within a day. Age at sampling was fitted as a covariate predictor in the models because not all nestlings in the two analyses were sampled at exactly 4 and 12 days of age. “Day 4” nestlings were sampled at a mean ± *SD* of 4.4 ± 1.02 days of age (range = 3–6), while “Day 12” nestlings were sampled at a mean ± *SD* of 11.7 ± 0.77 days of age (range = 10–14). Nestling mass was taken on the day of sampling, but due to fast growth during this period mass was adjusted to provide an expected mass for each nestling at either 4 or 12 days of age, using the slope from a linear model of mass on age for nestlings between 3 and 6, and 10 and 13 days of age, respectively. To calculate this adjustment, we used all relevant nestling mass measures available from our long‐term field study (day 4 mass adjustment slope = 2.81 g/day, *n* = 564 nestlings; day 12 mass adjustment slope = 1.92 g/day, *n* = 390 nestlings).

Sparrow‐weaver broods suffer relatively high levels of in‐nest predation (see Results). Accordingly, the majority of nestling disappearances in the model of survival from day 4 to the following breeding season occurred during the nestling period, when we have some information on the cause of disappearance. As the analyses above revealed that day 4 rtl predicted survival to the following season, we repeated this model with survival to fledging as the response and, having confirmed that the same relationship held, conducted the following tests with a view to teasing apart the contributions of predation‐ and starvation‐related mortality within the nest. Entire broods were categorized as having been “predated” when signs of predation were noted in the field following brood disappearance (specifically the presence of a hole in the back of the nest, which slender mongooses, *Galerella sanguinea*, have been observed to create when predating broods), or when all nestlings in multichick broods disappeared in the same interval between our standard brood survival checks (in total *n* = 14 nestlings from seven broods; all of which were in good condition prior to disappearance). Nestlings found dead within the nest without any sign of attempted predation, and nestlings that disappeared and left behind a heavier surviving sibling, were categorized as “expired” (*n* = 8 nestlings from 8 broods). We repeated model selection for the survival‐to‐fledging model twice: first with “predated” nestlings excluded from the full data set and second with “expired” nestlings excluded from the data set. The 10 nestlings (from 10 broods) that did not survive but did not fall into either the “predated” or “expired” categories were retained in both models.

#### 2.4.2 Does the within‐individual rate of change in telomere length predict survival?

We used binomial glmms (Bates et al., 2015) to test whether the within‐individual rate of change in RTL between days 4 and 12 of the nestling period predicted survival to the start of the following breeding season. We calculated rate of change in RTL as (day 12 RTL − day 4 RTL)/ number of days between the two sampling time points. As the rate of change in RTL could have nonlinear effects on downstream survival (e.g., differentially large effects of high rates of telomere loss), we included its quadratic effect. In addition, we included variables that the analyses above suggested were effective predictors of survival to the start of the following breeding season (specifically day 4 rtl, age at the start of the following breeding season, prelay rainfall, and day 12 body mass). The analysis utilized a sample size of 39 nestlings from 32 broods reared by 25 social groups over two breeding seasons. Of these 39 nestlings, 16 did not survive to the following breeding season, but we had no evidence that any had been predated within the nest. Of these 16, 10 are known to have survived to fledging. Social group and brood were fitted as random effects.

## 3 RESULTS

### 3.1 Does telomere length predict survival?

The Δ6 AICc top model sets from modelling the survival of 4‐day‐old and 12‐day‐old nestlings to the following breeding season are presented in Table 1. Counter to predictions, we found strong evidence that day 4 telomere length *negatively* predicts survival to the following breeding season, being present in both models in the top model set. The body mass of day 4 nestlings did not predict survival. Among day 12 nestlings, however, there was no evidence that telomere length predicted survival to the following breeding season, while body mass was an important positive predictor of survival. The relationships between telomere length (at both day 4 and day 12) and survival to the following breeding season are plotted in Figure S1. There was also evidence that prelay rainfall (and weaker evidence that postlay rainfall) negatively predicted survival. As expected, there was also strong evidence, for nestlings of both age classes, that the time lag between the focal nestling hatching and the start of the following breeding season (when their survival was assessed) negatively predicted survival (i.e., nestlings that hatched earlier in the breeding season were less likely to survive to the start of the following breeding season). There was no evidence for an effect of group size or the precise age at which the nestling was sampled on the survival of either day 4 or day 12 nestlings.

**Table 1:** Models investigating whether the telomere length (RTL) of (a) day 4 nestlings and (b) day 12 nestlings predict survival to the start of the following breeding season. The Δ6 AICc top model sets are shown relative to the null model (in grey). Effect sizes are given with standard errors in parentheses. Continuous variables were centred and scaled. Estimates for sex are given for males relative to females. Int = intercept, AW = adjusted weight after implementation of the model nesting rule (Richards et al., 2011). The same predictors were tested in both global models, with RTL, age at sampling and body mass being the values of these traits at day 4 or day 12 depending on the analysis

Int	RTL	Age next season	Body mass	Prelay rain	Postlay rain	Sex (M)	*df*	logLik	AICc	ΔAICc	AW
(a) Day 4 nestlings (*n* = 82 nestlings from 59 broods in 30 groups)											
−0.073	−1.063 (0.319)	−0.595 (0.274)					6	−48.56	110.2	0.00	0.814
−0.078	−0.843 (0.281)						5	−51.20	113.2	2.95	0.186
−0.027							4	−57.39	123.3	11.25	NA
(b) Day 12 nestlings (*n* = 146 nestlings from 100 broods in 36 groups)											
0.604		−2.164 (0.878)	0.695 (0.319)	−1.703 (0.730)	−0.596 (0.375)		8	−84.68	186.4	0.00	0.368
0.931		−1.933 (0.861)	0.931 (0.420)	−1.718 (0.814)		−0.915 (0.683)	8	−84.98	187.0	0.60	0.273
0.597		−1.733 (0.774)	0.642 (0.327)	−1.399 (0.646)			7	−86.14	187.1	0.69	0.260
0.783		−2.141 (0.807)		−1.419 (0.635)			6	−88.63	189.9	3.45	0.065
0.459			0.779 (0.311)				5	−90.36	191.2	4.75	0.034
0.427							4	−95.06	198.4	12.00	NA

Predictors included in the global model but absent from the top model set: age at sampling, adult group size.

**Figure 1:**
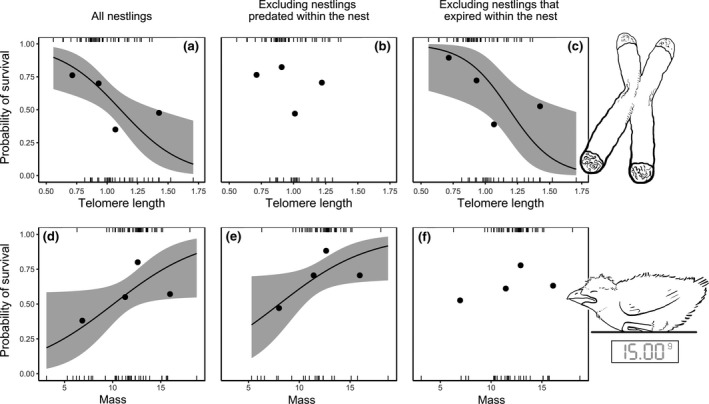
Model predictions with 95% confidence intervals for the effects of top: day 4 RTL (T/S) and bottom: body mass (g) on survival to fledging. Left panel: all nestlings. Central panel: all nestlings except those classified as “predated”. Right panel: all nestlings except those classified as “expired”. Points show average survival probabilities for each quartile, which were made for graphical representation only. Statistical analyses were based on individual values, shown with marks at 1 (survived) and 0 (not observed to have fledged). Where the variable of interest was present in the top model set, its effect is shown by the mean predicted line from the top model when all other variables are held at their mean value

That the relationship between telomere length and nestling survival to the following breeding season was apparent for day 4 nestlings but no longer evident at day 12 suggests that day 4 telomere length might predict short‐term survival during the nestling period. Accordingly, additional modelling revealed that telomere length at day 4 strongly negatively predicted survival to *fledging*, being present in all models in the Δ6 AICc top model set (Figure 1a; Table 2a). There was also strong evidence that body mass at day 4 *positively* predicted survival to fledging (Figure 1d; Table 2a). Attempts to tease apart the relative contributions of predation and other sources of mortality to this counterintuitive relationship between day 4 telomere length and survival suggest that broods with longer telomeres suffered higher in‐nest predation but were no more likely to die from other causes. Specifically, the removal of “predated” broods (see Methods) from this survival‐to‐fledging data set (14 of the 32 nestlings that did not survive to fledging) resulted in a Δ6 AICc top model set that now provided no support for telomere length as a predictor (Figure 1b; Table 2b), retaining only a positive effect of nestling mass (Figure 1e; Table 2b). By contrast, the removal of “expired” nestlings from the survival‐to‐fledging data set (8 of the 32 nestlings that did not survive to fledging) resulted in a Δ6 AICc top model that retained the negative effect of telomere length (Figure 1c; Table 2c) but now lacked support for nestling mass as a predictor (Figure 1f; Table 2c). Together, these findings suggest that day 4 telomere length principally predicted mortality arising from in‐nest predation, while mortality following the exclusion of known predation was instead explained by variation in nestling mass.

**Table 2:** Models investigating whether day 4 nestling RTL predicts survival to fledging, for (a) all samples, (b) when “predated” nestlings were excluded and (c) when “expired” nestlings were excluded. The Δ6 AICc top model sets are shown relative to the null model (in grey). Effect sizes are given with standard errors in parentheses. All continuous variables were centred and scaled. Estimates for sex are given for males relative to females. Int = intercept, AW = adjusted weight after implementation of the model nesting rule (Richards et al., 2011). The same predictors were tested in all global models

Intercept	RTL	Body mass	Sex (M)	Group size	Postlay rain	*df*	logLik	AICc	Δ AICc	AW
(a) All samples (*n* = 82 nestlings from 59 broods from 30 groups)										
0.354	−0.825 (0.316)	0.578 (0.295)		−0.424 (0.259)		7	−47.30	110.1	0.00	0.296
0.381	−0.888 (0.334)	0.686 (0.335)			−0.431 (0.293)	7	−47.42	110.4	0.24	0.262
0.367	−0.862 (0.327)	0.560 (0.307)				6	−48.66	110.4	0.33	0.251
−0.055	−0.845 (0.302)		0.760 (0.502)			6	−49.62	112.4	2.24	0.097
0.329	−0.804 (0.300)					5	−50.81	112.4	2.30	0.094
0.383						4	−56.15	120.8	9.99	0.002
(b) Excluding “predated” nestlings (leaves *n* = 68 nestlings from 52 broods from 30 groups)										
0.859		0.571 (0.287)				5	−39.86	90.7	0.00	0.733
0.806						4	−42.04	92.7	2.01	0.267
(c) Excluding “expired” nestlings (leaves *n* = 74 nestlings from 55 broods from 30 groups)										
0.941	−1.229 (0.533)				−0.673 (0.479)	6	−40.21	93.7	0.00	0.51
8.242						4	−42.59	93.8	0.08	0.49

Predictors included in the global model for (a) but absent from the top model set: age at sampling, prelay rain.

### 3.2 Does the within‐individual rate of change in telomere length predict survival?

**Figure 2:**
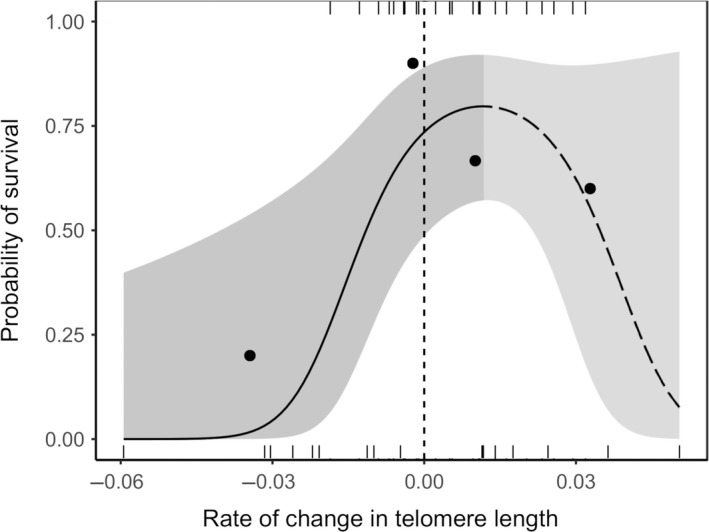
Model predicted line with 95% confidence interval showing the quadratic relationship between a nestling's rate of change in RTL (T/S) per day between days 4 and 12 and their downstream survival to the start of the following breeding season (from Table 3a). Points show average survival probabilities for each quartile, which were made for graphical representation only. Statistical analyses were based on individual values, shown with marks at 1 (survived) and 0 (not observed the following season). Points to the left of the vertical dashed line indicate reductions in mean telomere length, while points to the right indicate increases in mean telomere length. The regression line presents the mean predicted relationship when body mass is held at its mean value. The regression line is solid prior to the peak of the quadratic as there is a significant positive relationship between the rate of change in telomere length and survival in this half of the data set (Table 3b). The regression line is dashed after the peak of the quadratic as there is no significant relationship in this half of the data set (there is no evidence of reduced survival among offspring that experience larger *increases* in mean telomere length; Table 3c)

As predicted, the within‐individual rate of change in telomere length between day 4 and day 12 of the nestling period predicted a nestling's probability of survival to the following breeding season (Table 3a). A positive linear effect of the rate of change in telomere length was present in the top three models, but our findings suggest that a quadratic relationship best explains the data, as a negative effect of the square of the rate change in telomere length was also present in the top two models (Table 3a; Figure 2). This relationship was robust to the exclusion of a possible outlier in the rate of change in telomere length data (rate of change in RTL = −0.06, which was more than twice the interquartile range below the first quartile of the data set), with the linear and quadratic terms both retained within the top two models (Appendix S3.3). Models containing the “telomere length at day 4” predictor were not present among the top three models (Table 3a), and the addition of this predictor to the top model did not qualitatively change the effect size estimates for the “change in telomere length” predictors (Table 3a, model included in italics). There was no evidence that in‐nest predation (see Methods) was the cause of death for any of the 16 nestlings in this analysis that failed to survive to the following breeding season. We provide plots of the within‐individual telomere length slopes from day 4 to day 12 for individuals that did and did not survive to following breeding season, in Figure S2.

**Table 3:** Models investigating whether a nestling's rate of change in telomere length (ΔRTL) from day 4 to day 12 predicts their survival to the start of the following breeding season, for (a) all data, (b) for ΔRTL data below the value that yields the peak of the quadratic and (c) for ΔRTL data above the value that yields the peak of the quadratic. The Δ6 AICc top model sets are shown relative to the null model (shown in grey). The best performing model that contained day 4 RTL is included in italics for reference (though was excluded from the top model set following the nesting rule; Richards et al., 2011) Effect sizes are given with standard errors in parentheses. Continuous variables were centred and scaled. Int = intercept, AW = adjusted weight. Where variables were not present in the global model NAs are given

Int	Day 12 body mass	Day 4 RTL	ΔRTL	ΔRTL^2^	*df*	logLik	AICc	ΔAICc	AW
(a) All data (*n* = 39)									
1.150	1.007 (0.573)		1.036 (0.605)	−1.180 (0.631)	6	−18.337	51.3	0.00	0.496
1.366			0.747 (0.516)	−1.351 (0.574)	5	−20.256	52.3	1.03	0.296
0.306	1.299 (0.554)		0.862 (0.446)		5	−21.199	54.2	2.92	0.115
*1.148*	*0.996 *(*0.581*)	*−0.057 *(*0.524*)	*0.986 *(*0.754*)	*−1.172 *(*0.631*)	*7*	*−18.33*	*54.3*	*2.98*	*NA*
0.381	0.981 (0.584)	−0.688 (0.472)			5	−22.099	56.0	4.72	0.047
0.358	0.903 (0.430)				4	−23.451	56.1	4.78	0.045
0.363					3	−26.401	59.5	8.19	NA
(b) Data below the peak of the quadratic (*n* = 26)									
0.533			2.033 (0.828)	NA	2	−11.688	27.9	0.00	1.00
*0.547*		*−0.191 *(*0.602*)	*1.900 *(*0.908*)	*NA*	*3*	*−11.64*	*30.37*	*2.47*	*NA*
0.470				NA	2	−17.323	36.8	8.92	NA
(c) Data above the peak of the quadratic (*n* = 13)									
0.1542				NA	3	−8.972	26.6	0.00	1.00

Predictors included in the global model for (a) but absent from the top model set: day 4 RTL, age at the start of the following breeding season, prelay rainfall, postlay rainfall.

Given the support for a quadratic relationship between rate of change in telomere length and survival, we split the data at the value corresponding to the peak of the quadratic curve (rate of change in telomere length per day = 0.012) and repeated the model comparison process (a) for data with rate values *below* the peak (to establish whether there was evidence of a *positive* association between the rate of change in telomere length and survival in these data) and (b) for data with rate values *above* the peak (to establish whether there was evidence of a *negative* association between the rate of change in telomere length and survival in these data). In each case, we constructed a global model that only included terms present in the top model set derived using the entire data set (see Table 3a) and compared all possible nested models. We were unable to fit brood or social group as random effects for the data below the peak, due to convergence errors, and so used a linear model with no random effects in this case. This approach revealed support in data below the peak of the quadratic for a positive linear effect of the rate of change in telomere length on survival to the following season (Table 3b). This cannot be attributed to a failure to control for brood as a random factor, as rerunning the analysis utilizing just one nestling per brood (for the two broods that contained more than one nestling) yielded the same result for all possible combinations of retained nestlings. By contrast, in the data above the peak of the quadratic there was no evidence for an effect of the rate of change in telomere length on survival (Table 3c; we verified that this remained the case even after random effects were removed). These patterns are consistent with the negative effect of the quadratic term in the full data set reflecting an asymptote in the increase in survival (rather than a reduction in survival) as the rate of change in telomere length increases beyond zero. Comparable results were obtained when splitting the data set around the point of zero within‐individual change in telomere length (Appendix S3.4), indicating an association between telomere attrition and survival among individuals experiencing an apparent decrease in telomere length but not among those experiencing an apparent increase in telomere length.

## 4 DISCUSSION

To shed light on the relative utility of telomere length and within‐individual telomere attrition in early life as biomarkers of SI, we investigated the extent to which both these telomeric traits predict downstream survival in the wild. Contrary to expectations, telomere length soon after hatching negatively predicted survival. Our analyses suggest that this effect arises because broods of hatchlings with longer telomeres are more likely to suffer in‐nest predation (Figure 1c). There was no evidence to suggest that they were differentially vulnerable to other sources of in‐nest mortality (Figure 1b). Concordant with our predictions, however, nestlings that experienced higher rates of telomere attrition were less likely to survive to the following breeding season (Figure 2). This relationship appeared to be asymptotic, such that larger within‐individual decreases in telomere length predicted reduced survival (Figure 2, left), whereas the magnitude of within‐individual increases in telomere length (Figure 2, right) did not predict survival. These findings reveal added complexity in the nature of associations between telomeric traits and performance, and highlight the potential benefits of complementing telomere length measures with longitudinal assessments of telomere attrition since (or within) early life. Below we consider likely explanations for our findings as well as their wider implications for future work seeking to utilize telomeric traits to study the causes and consequences of variation in somatic maintenance and integrity.

Several potential explanations exist for the unexpected negative association between day 4 telomere length and survival to fledgling (Figure 1a). Hatchlings investing more heavily in somatic maintenance could consequently have fewer reserves available to weather short‐term resource shortages as nestlings, leaving them differentially vulnerable to starvation (see McLennan et al., 2016 for similar logic). However, that this survival association was no longer apparent when predated broods were excluded from the analysis (Figure 1b) and remained apparent when nestlings that likely starved were excluded (Figure 1c) suggests that broods of nestlings with longer telomeres may have been differentially vulnerable to in‐nest predation, rather than starvation. While this association with nest predation risk could reflect a chance occurrence, at least two plausible explanations exist for it. First, environmental conditions that yield broods of hatchlings with longer telomeres could also increase nest predation risk. For example, prelaying rainfall positively predicts hatchling telomere length in this species (Wood, 2017), and rainfall could also increase nest predation risk if predators were rain‐dependent breeders and/or tracked hot‐spots of recent rainfall (Jaksić et al., 1993; Yang, Bastow, Spence, & Wright, 2008). It seems unlikely that this precise mechanism accounts for our findings, however, as variation in prelaying rainfall was controlled in the survival analyses presented and did not predict day 4 nestling survival. Second, and more plausibly, broods of hatchlings with longer telomeres could simply be of higher intrinsic quality and may therefore have begged harder during the nestling period (Kilner, 2001), leaving them more likely to attract nest predators (Leech & Leonard, 1997).

While this inverse relationship between telomere length and survival provides no support for the hypothesis that among‐individual variation in telomere length is a useful biomarker of SI, it could nevertheless be reconciled with this hypothesis being correct (e.g., via the scenarios outlined above). That said, two of our other findings also question the utility of telomere length as a biomarker of SI in this context: among‐individual variation in telomere length failed to predict (a) the survival of day 4 nestlings to fledging when predated broods were removed (Figure 1b) and (b) the survival of day 12 nestlings to the following breeding season (Figure S1b). One potential explanation for this suite of findings is that among‐individual variation in telomere length *is* an effective biomarker of SI, but SI is not relevant to first‐year survival. Alternatively, telomeric traits could simply be unrelated to SI in this species. However, our key finding that telomere attrition (between days 4 and 12) *does* predict downstream survival to the following breeding season points instead to a third explanation. Telomere attrition could effectively capture declines in SI, but fail to manifest as an evident relationship between telomere length and SI (and hence survival) due to unrelated sources of among‐individual variation in telomere length (see Boonekamp et al., 2014 for similar logic). For example, among‐individual variation in telomere length, particularly in early life, could be a product in part of genetic and/or epigenetic sources of variation in “initial” telomere length at conception and (in studies such as ours that assess telomere length using qPCR) among‐individual variation in the incidence of interstitial telomeric repeats (Delany et al., 2003; Dugdale & Richardson, 2018; Eisenberg & Kuzawa, 2018). Indeed, while such sources of variation in telomere length could conceivably obscure relationships between telomere length per se and survival, it is also worth noting their potential to artificially generate such relationships (if a source of variation in an offspring's “initial” telomere length independently impacts their downstream survival, such as might be imagined for parental age effects).

Our finding that higher rates of telomere attrition negatively predict survival follows expectations regarding the negative fitness consequences of declines in SI and lends strength to the view that longitudinal assessments of telomere attrition since (or within) early life may constitute a powerful biomarker of SI (Boonekamp et al., 2014). Notably, this relationship is apparent even after allowing for positive effects of body mass on downstream survival (Figure 2). Our findings concord with those of the only other study to date to have investigated relationships between both telomere length and attrition rate in free‐living nestlings and their downstream survival, in which jackdaw telomere attrition but not length predicted survival to recruitment (Boonekamp et al., 2014). Higher telomere attrition rates could predict poorer future survival prospects through either of the following mechanisms. First, faster telomere attrition may hasten the accumulation of senescent cells and the depletion of stem cell stocks (Blasco, 2007; Boonekamp et al., 2013; Herbig et al., 2006), thereby accelerating tissue degeneration and compromising organismal survival prospects (Campisi & D'Adda Di Fagagna, 2007; Coppé, Desprez, Krtolica, & Campisi, 2010). Second, higher telomere attrition rates may effectively act as a biomarker of accumulated damage to other tissues across the body that may itself have causal downstream effects on fitness (Metcalfe & Alonso‐Alvarez, 2010; Selman, Blount, Nussey, & Speakman, 2012; Young, 2018). In both scenarios, telomere attrition is envisaged to provide a biomarker of declines in SI, and declines in SI are considered the causal agent in the survival outcome. It is worth noting, however, that such declines could predict deficits in downstream performance without actually causing them. For example, the environment that compromised SI during the telomere measurement window could (a) carry forward in time to independently impact downstream survival prospects or (b) impact other aspects of internal state (such as aspects of immunity or resource reserves) that could themselves impact downstream survival prospects. While our statistical models seek to control for such direct effects of a nestling's social and abiotic (rainfall) environment, as well as its body mass, it nevertheless remains possible (both in this and all other such studies to date) that the survival relationship detected is not actually caused by deficits in SI. Indeed, challenges with the assessment of SI at the organismal level and the elucidation of its causal effects leave it currently unclear to what extent variation in SI actually is responsible for variation in performance in early life.

Uniquely, to our knowledge, our analyses revealed a nonlinear association between telomere attrition rate and survival: faster rates of telomere *attrition* among nestlings predicted reduced survival, but faster rates of telomere *elongation* among nestlings did not predict increased survival (Figure 2). While our analyses suggest that many nestlings could have experienced telomere elongation between days 4 and 12, such a pattern could arise from mechanisms such as the drop out over time of haematopoetic stem cell lineages with shorter telomeres or indeed measurement error (Steenstrup, Hjelmborg, Kark, Christensen, & Aviv, 2013; though see Bateson & Nettle, 2017). Mechanisms of this kind should weaken relationships between within‐individual changes in telomere length and SI (and hence downstream survival), which could explain the lack of an association with survival among those nestlings apparently experiencing increases in telomere length. That said, it is also possible that the observed within‐individual increases in telomere length reflect at least some degree of true telomere elongation, as telomerase activity (which can extend telomeres) has been reported in the bone marrow of nestlings in several avian species (Haussmann, Winkler, Huntington, Nisbet, & Vleck, 2004, 2007). The extent to which telomerase activity may weaken the utility of within‐individual telomere dynamics (and, indeed, the utility of telomere length) as a biomarker of SI is unclear, as telomere repair by telomerase may itself be inhibited by oxidative damage (Ahmed et al., 2008), and telomerase has also been shown to have restorative impacts on structures other than telomeres (Ahmed et al., 2008; Fu et al., 2000; Sharma et al., 2003). Indeed, the nonlinear relationship between change in telomere length and survival could still be consistent with change in telomere length accurately capturing changes in SI, if only marked reductions in SI impact survival in the first year of life. Given the variation among species in the extent to which telomere elongation is suppressed (Haussmann, Winkler, Huntington, Nisbet, & Vleck, 2007; Seluanov et al., 2007), the impact of telomere elongation mechanisms on the utility of telomeric traits as a biomarker of SI would be a useful line of future investigation.

Together, our findings support the growing use of telomeric traits as a biomarker of variation in SI and lend strength to the view that in some contexts SI may be more effectively captured via longitudinal assessments of rates of change in telomere length since early life than single measures of telomere length in isolation (Boonekamp et al., 2014). As telomeric traits have now frequently been found to predict aspects of downstream performance (Table S1; Wilbourn et al., 2018), establishing the causality of such relationships has become a pressing challenge for the field, with two key issues demanding attention. First, whether telomeric traits play a causal role in downstream performance deficits or act principally as a biomarker of wider deficits in SI (Simons, 2015; Young, 2018). Second, whether deficits in SI play a causal role in compromised downstream performance, given the potential for factors correlated with deficits in SI to themselves causally impact downstream performance (e.g., reduced elaboration of key fitness‐relevant organs, such as the immune system, regardless of the extent of biomolecular errors or damage therein). While the former challenge is a key focus of interest in telomere biology (Criscuolo, Smith, Zahn, Heidinger, & Haussmann, 2018; Simons, 2015; Young, 2018), the latter may ultimately prove the more significant objective for those with a wider interest in life history trade‐offs and their impacts on ageing trajectories.

## AUTHOR CONTRIBUTIONS

Both authors designed the study. E.M.W. led the fieldwork and both authors contributed to sample collection. E.M.W. performed laboratory and data analyses and wrote the manuscript. Both authors edited the manuscript and approved the final version.

## Data Availability

Data analysed for this study are available at https://github.com/EmmaMWood/p.mahali_survival.
