# Supplementary material for: Telomere attrition predicts reduced survival in a wild social bird, but short telomeres do not
Source: Mol Ecol. 2019 Aug 7;28(16):3669–80. doi: 10.1111/mec.15181 (PMC6772082; doi:10.1111/mec.15181)
Supplement: Supplementary file 1 [file MEC-28-3669-s001.doc]

**Supplemental Information for:**

**Telomere attrition predicts reduced survival in a wild social bird,**

**but short telomeres do not**

Emma M. Wood & Andrew J. Young

Table of Contents:

| **S1 Table of studies** | Page 2 |
| --- | --- |
| **S2 Supplementary methods**  S2.1 Molecular sexing  S2.2 Assessment of DNA integrity and purity  S2.3 Calculation of RTL and inter-plate repeatability | Page 4  Page 4  Page 5 |
| **S3 Supplementary results**  S3.1 Supporting figures:   - Figure S1, effects of RTL and mass on survival to the following season - Figure S2, RTL slopes between day 4 and day 12   S3.2 Full Δ6 AICc tables prior to model nesting rule, for:   - Table 1a - Table 1b - Table 2a - Table 2b - Table 2c - Table 3a - Table 3b - Table 3c | Page 6  Page 7  Page 8  Page 10  Page 12  Page 14  Page 16  Page 18  Page 18  Page 19 |
| S3.3 Effect of outlier removal on model of within-individual rate of  change in telomere length on survival | Page 19 |
| S3.4 Models of survival to the following season for individuals showing  a) a decrease and b) an increase in RTL between 4 and 12 days of age | Page 20 |
| **References** | Page 21 |

**S1 Table of studies**

**Table S1** – Studies of wild vertebrates investigating whether natural variation in telomere length or telomere attrition rate predicts survival. Studies are grouped first by whether they focussed on dependent young or adults, and then by taxonomic group. Telomere length: ‘Yes’ where shorter telomeres predict shorter lifespan and/or reduced survival. Telomere attrition: ‘Yes’ where higher rates of telomere attrition predict shorter lifespan and/or reduced survival. Sample sizes (n; number of individuals) are given in parentheses. * only in nestlings that never contracted malaria; sample size is given for uninfected birds only. Infected birds: n = 32. † Only in late-born chicks though sample size is given for all chicks. ‡ Pythons that were recaptured had shorter telomeres than those that were not.

| **Species** | **Survival measure** | **Telomere length (n)** | **Telomere attrition (n)** | **Reference** |
| --- | --- | --- | --- | --- |
| **Studies of dependent young** | | | | |
| **Great reed warbler (*Acrocephalus arundinaceus*)** | lifespan | **Yes ***(49) | - | Asgha*r et a*l. 2015 |
| **Jackdaw (*Corvus monedula*)** | survival to post-fledging and to recruitment | **No** (152) | **Yes** (152) | Boonekam*p et a*l. 2014 |
| **Barn swallow (*Hirundo rustico*)** | lifespan | **No** (60) | - | Capriol*i et a*l. 2013 |
| **Magellanic penguin (*Spheniscus magellanicus*)** | Survival to fledging | **No** (20) | - | Cerchiar*a et a*l. 2017 |
| **King penguin (*Aptenodytes patagonicu*s)** | growth-period survival | **Yes** (44) | - | Geige*r et a*l. 2012 |
| **Great tit (*Parus major*)** | survival to recruitment | **Yes** (327) |  | Salmó*n et a*l. 2017 |
| ***Parus major*** | post-recruitment survival | **Yes** (118) |  | Salmón *et al.* 2017 |
| ***Aptenodytes patagonicus*** | survival to fledging  survival to winter | **Yes** ^†^ (66)  **No** (63) | -  - | Stie*r et a*l. 2014 |
| **European storm petrel (*Hydrobates pelagicus*)** | survival to fledging | **Yes** (59) | - | Watso*n et a*l. 2015 |
| **Black legged kittiwake (*Rissa tridactyla*)** | survival to fledging | **Yes** (107) | - | Youn*g et a*l. 2017 |
| **Soay sheep (*Ovis aries*) female neonates** | first winter survival | **No**  (n unclear) |  | Fairli*e et a*l. 2016 |
| ***Ovis aries* female lambs in their 1st August** | first winter survival | **Yes** (115) | **No**  (n unclear) | Fairli*e et a*l. 2016 |
| **Water python (*Liasis fuscus*)** | recapture | **No** (20) | - | Ujvari & Madsen 2009 |
| **Studies of adults** | | | | |
| **American redstart (*Setophaga ruticilla*) ♂** | return rate | **Yes** (63) | - | Angelie*r et a*l. 2013 |
| **Seychelles warbler (*Acrocephalus sechellensis*)** | survival to next year  post-sampling lifespan | **Yes** (204)  **Yes** (204) | -  **Yes** (96) | Barret*t et a*l. 2013 |
| **Alpine swift (*Apus melba*)** | annual survival over 7 years | **Yes** (98) | **Yes** (22) | Biz*e et* al 2009 |
| **Southern giant petrel (*Macronectes giganteus*)** | survival over 8 years | **Yes** (47) | - | Foot*e et a*l. 2010 |
| **Tree swallow (*Tachycineta bicolor*)** | return rate | **Yes** (22) | - | Haussman*n et a*l. 2005 |
| ***Tachycineta bicolor*** | return rate over 2 years | **No** (82) | - | Ouyan*g et a*l. 2016 |
| **Sand martin (*Riparia riparia*)** | age last seen | **Yes** (23) | - | Paulin*y et a*l. 2006 |
| **Dunlin (*Calidris alpine*)** | minimum lifespan  (age last seen) | **No** (30) | - | Pauliny *et al.* 2006 |
| **Jackdaw (*Corvus monedula*)** | survival to following year | **Yes** (48 adults + 74 nestlings) | **Yes**  (n unclear) | Salomon*s et a*l. 2009 |
| **Yellowthroat (*Geothlypis trichas*) ♂** | survival to next year | **No** (59) | **No** (47) | Taff & Freeman-Gallant 2017 |
| **Water python (*Liasis fuscus*)** | recapture | **No** ^‡^ (50) | - | Ujvari & Madsen, 2009 |
| **Frill-necked lizard (*Chlamydosaurus kingie*)** | recapture | **No** (93) | **No** (40) | Ujvar*i et a*l. 2016 |
| **Soay sheep (*Ovis aries*) females >3 years old** | Lifespan and survival  Survival over high -mortality winter | **No** (216)  **Yes** (86) |  | Fairlie *et al.* 2016 |

**S2 Supplementary methods**

**S2.1 Molecular sexing**

The 87 nestlings that did not survive to the following season (precluding them being sexed on the basis of morphological characters) were molecularly sexed using the Z-002A primers (Dawson 2007). The PCR mix contained 5uL Q mix, 1uL FZ-002A, 1uL RZ-002A, 1 uLDNA at approximately 10ng/uL, and 2uL ddH2O. The following PCR conditions were used: An initial step of 94°C for 3 minutes, followed by 30 cycles of 94°C for 45 seconds, 48°C for 1 minute and 72°C for 1 minute. The final step was 72°C for 10 minutes. The PCR product was resolved on a 3% gel.

**S2.2 DNA extraction and assessment of DNA integrity and purity**

For DNA extraction, the manufacturer’s protocol for whole blood was followed, with the following exceptions: bird blood is nucleated, so red cells were not separated. 225uL of lysis solution was used instead of 3000uL. An overnight lysis step at 55°C was required. After the protein precipitation solution was added, the mixture was left on ice for 10 minutes, and was centrifuged for 6 minutes rather than 3 minutes. Finally, to ensure high concentration DNA, 20uL of DNA hydration solution was added to the DNA pellet, rather than 100uL. DNA concentration was then measured, and further dilutions made as required.

We assessed DNA integrity of all sample extracts by gel electrophoresis, accepting only samples with good integrity (those where clear crowns were preset, and smearing absent). We measured DNA concentration and purity on a NanoVue 4282 Spectrophotometer (v1.7.3) and accepted ratios of between 1.7 and 2.0 for 260/280, and between 1.9 and 2.2 for 260/230. Where there was enough sample, those with poor integrity or purity were re-extracted. If the second extraction also fell below the required quality, the sample was discarded. 14 samples were discarded due to poor purity. No samples were discarded due to poor integrity.

**S2.3 Calculation of RTL and inter-plate repeatability**

DNA pooled from three individuals was used to create a 2x serial dilution from 10ng to 0.625ng, which was run in triplicate on each plate to assess reaction efficiencies (GAPDH efficiency: 90.6-110.4, r^2^ = 0.99-1.00, telomere efficiency: 85.8-109.4, r^2^ = 0.99-1) and to ascertain that sample measurements had been taken within the linear phase of amplification. The 5ng dilution from the standard curve was used as a between-plate calibration sample to standardise across plates. GAPDH and telomere reactions were carried out on separate 96-well plates on a Stratagene Mx3000 instrument. Samples were run on 22 plate pairs (alongside samples to be used for analyses in other manuscripts), with samples from the same individual run on the same plate. We imported background-corrected data into LinRegPCR (Ruijte*r et a*l. 2009) to correct baseline fluorescence, set constant fluorescence thresholds within the windows of linearity for GAPDH (0.156) and telomere (0.161), and to calculate individual well efficiencies.

We exported Cq values (the cycle at which fluorescence crosses the constant fluorescence threshold) and well efficiencies from the LinReg output into Excel, where we calculated the mean and coefficient of variation of Cq and efficiency for each triplicate. We calculated mean well efficiency for each plate, excluding well efficiencies outside the 5^th^ and 95^th^ percentiles. Mean reaction efficiencies were between 1.89 and 1.93 for GAPDH plates, and between 1.77 and 1.84 for telomere plates. Triplicate mean efficiencies that were different to the mean plate efficiency for their plate by more than 5% and triplicates for which the Cq %CV was greater than 5% were highlighted and examined individually. Reactions that were clear outliers within their triplicate were excluded from the triplicate and mean Cq and efficiency was recalculated from the remaining reactions. Triplicates for which there was no clear outlier were excluded from further analysis. Samples that fell outside the range of the standard curve were also excluded from further analysis. Mean Cq was 15.0 for telomere, and 25.9 for GAPDH reactions.

RTL was calculated as the ratio of the quantity of telomere to control gene (T/S) according to Pfaffl (2001) using the following equation, where E_TEL_ and E_GAPDH_ are the mean plate well efficiencies for telomeres and GAPDH respectively:

RTL = (E_TEL_ ^ (Cq_TEL[Calibrator]_ – Cq_TEL[Sample]_)) / (E_GAPDH_ ^ (Cq_GAPDH[Calibrator]_ – Cq_GAPDH [Sample]_))

Samples for further studies were run alongside those used here. We calculated inter-plate repeatability from 141 samples that were run on at least 2 plates, which included samples used in further studies; these were 13 adult and 12 nestling samples on three ‘repeatability’ plates, 1 adult sample run on 16 plates, and a further 114 samples (109 from adults, and 5 from nestlings) run on 28 plates. Inter-plate co-efficient of variation of RTL was 13.09% (SD = 8.15). Inter-plate co-efficient of variation of just the 17 nestling samples was 13.62% (SD = 8.15).

**S3 Supplementary results**

**S3.1 Supplementary plots**

**
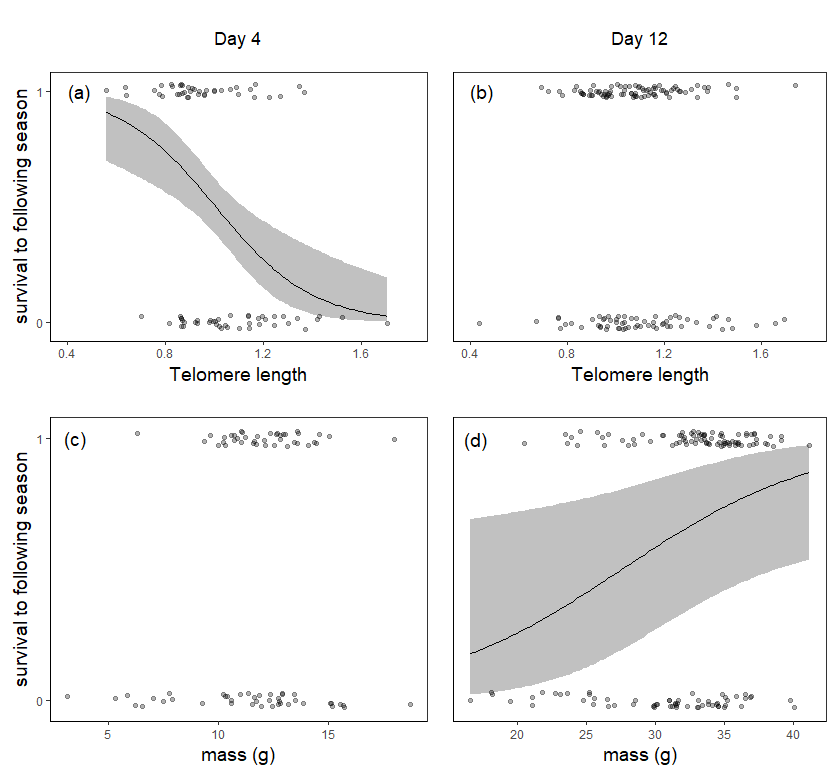
**

**Figure S1**. Model predictions with 95% confidence intervals for the effects of a) day 4 RTL (T/S), b) day 12 RTL, c) day 4 mass (g) and d) day 12 mass (g) on survival to the following season. Where survival = 0 the nestling was never observed in the following season, or at any point after, where survival = 1 the nestling was observed in the following season. Points are jittered raw data. Where the variable of interest was present in the top model set, its effect is shown by the mean predicted line and 95% confidence intervals from the top model when all other variables are held at their mean value.


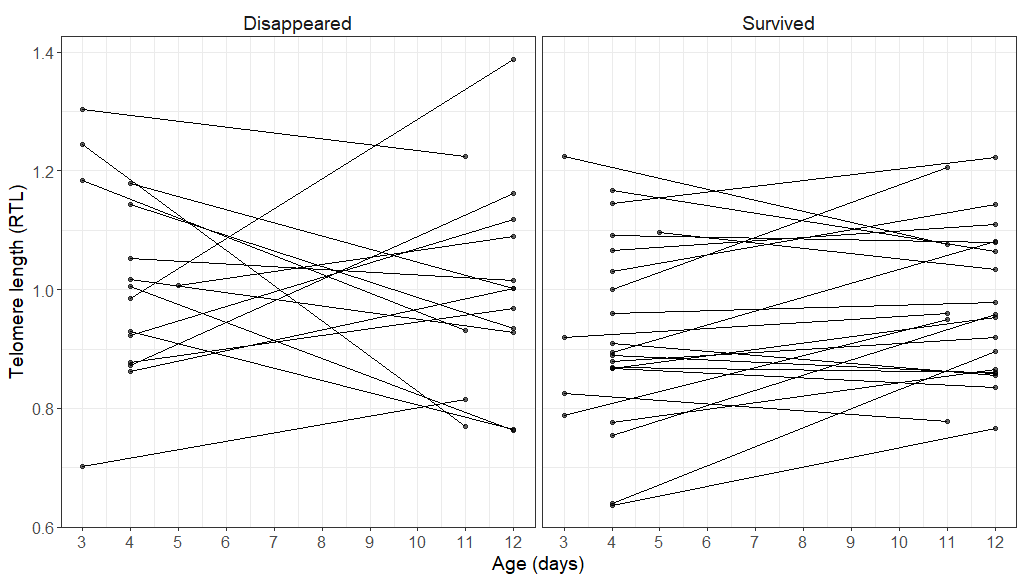


**Figure S2**. Slopes of change in telomere length between day 4 (+/- 1 day) and day 12 (+/- 1 day) nestlings. Left: nestlings that were not seen to have survived to the following season. Right: Nestlings that survived to the following season.

**S3.2 Full Δ6 AICc model sets prior to implementation of model nesting rule**

**Full model set for Table 1a:** Day 4 nestling survival to the start of the following season.

Effect sizes are given for centered and scaled continuous variables. Presence of factors in the model is indicated with +.

| Intercept | Sex | Age | Age next season | Body mass | Group Size | post- lay rain | pre- lay rain | RTL | df | logLik | AICc | Delta AICc | weight |
| --- | --- | --- | --- | --- | --- | --- | --- | --- | --- | --- | --- | --- | --- |
| -0.073 |  |  | -0.595 |  |  |  |  | -1.063 | 6 | -48.56 | 110.24 | 0.00 | 0.105 |
| -0.060 |  |  | -0.684 |  |  | -0.304 |  | -1.111 | 7 | -47.83 | 111.16 | 0.92 | 0.066 |
| -0.069 |  |  | -0.528 |  | -0.231 |  |  | -1.043 | 7 | -48.16 | 111.83 | 1.59 | 0.048 |
| -0.075 |  |  | -0.806 |  |  |  | -0.300 | -1.026 | 7 | -48.26 | 112.04 | 1.80 | 0.043 |
| -0.211 | + |  | -0.571 |  |  |  |  | -1.067 | 7 | -48.41 | 112.33 | 2.09 | 0.037 |
| -0.073 |  |  | -0.566 | 0.074 |  |  |  | -1.051 | 7 | -48.52 | 112.55 | 2.31 | 0.033 |
| -0.071 |  | -0.047 | -0.611 |  |  |  |  | -1.058 | 7 | -48.55 | 112.60 | 2.36 | 0.032 |
| -0.063 |  |  | -0.944 |  |  | -0.330 | -0.357 | -1.072 | 8 | -47.42 | 112.82 | 2.58 | 0.029 |
| -0.072 |  | 0.354 | -0.644 |  |  | -0.529 |  | -1.206 | 8 | -47.43 | 112.83 | 2.59 | 0.029 |
| -0.059 |  |  | -0.623 |  | -0.212 | -0.291 |  | -1.092 | 8 | -47.50 | 112.98 | 2.74 | 0.027 |
| -0.242 | + |  | -0.657 |  |  | -0.327 |  | -1.119 | 8 | -47.58 | 113.12 | 2.88 | 0.025 |
| -0.078 |  |  |  |  |  |  |  | -0.843 | 5 | -51.20 | 113.19 | 2.95 | 0.024 |
| -0.083 |  |  |  |  | -0.367 |  |  | -0.851 | 6 | -50.05 | 113.22 | 2.98 | 0.024 |
| -0.061 |  |  | -0.641 | 0.133 |  | -0.328 |  | -1.098 | 8 | -47.71 | 113.39 | 3.15 | 0.022 |
| -0.081 |  | 0.558 | -1.017 |  |  | -0.689 | -0.587 | -1.160 | 9 | -46.52 | 113.55 | 3.31 | 0.020 |
| -0.069 |  |  | -0.722 |  | -0.213 |  | -0.266 | -1.011 | 8 | -47.93 | 113.84 | 3.60 | 0.017 |
| -0.077 |  |  |  | 0.313 | -0.405 |  |  | -0.853 | 7 | -49.24 | 114.00 | 3.76 | 0.016 |
| -0.068 |  |  | -0.463 | 0.137 | -0.263 |  |  | -1.018 | 8 | -48.03 | 114.03 | 3.79 | 0.016 |
| -0.172 | + |  | -0.515 |  | -0.213 |  |  | -1.047 | 8 | -48.08 | 114.13 | 3.89 | 0.015 |
| -0.077 |  |  |  |  |  |  | 0.304 | -0.966 | 6 | -50.55 | 114.22 | 3.98 | 0.014 |
| -0.208 | + |  | -0.778 |  |  |  | -0.295 | -1.030 | 8 | -48.13 | 114.23 | 3.99 | 0.014 |
| -0.070 |  | 0.008 | -0.524 |  | -0.233 |  |  | -1.044 | 8 | -48.16 | 114.29 | 4.05 | 0.014 |
| -0.077 |  |  |  | 0.273 |  |  |  | -0.856 | 6 | -50.60 | 114.31 | 4.07 | 0.014 |
| -0.076 |  |  | -0.781 | 0.107 |  |  | -0.325 | -1.005 | 8 | -48.18 | 114.34 | 4.10 | 0.014 |
| -0.071 |  | 0.428 | -0.536 |  | -0.272 | -0.547 |  | -1.182 | 9 | -46.93 | 114.36 | 4.12 | 0.013 |
| -0.075 |  | 0.003 | -0.806 |  |  |  | -0.301 | -1.026 | 8 | -48.26 | 114.50 | 4.26 | 0.013 |
| -0.079 |  |  |  |  | -0.339 |  | 0.255 | -0.947 | 7 | -49.59 | 114.70 | 4.46 | 0.011 |
| -0.215 | + | -0.064 | -0.592 |  |  |  |  | -1.060 | 8 | -48.38 | 114.73 | 4.50 | 0.011 |
| -0.205 | + |  | -0.549 | 0.058 |  |  |  | -1.058 | 8 | -48.38 | 114.74 | 4.50 | 0.011 |
| -0.287 | + |  |  |  |  |  |  | -0.863 | 6 | -50.83 | 114.78 | 4.54 | 0.011 |
| -0.094 |  | 0.622 |  |  | -0.433 | -0.554 |  | -1.011 | 8 | -48.44 | 114.86 | 4.62 | 0.010 |
| -0.244 | + |  | -0.913 |  |  | -0.354 | -0.355 | -1.080 | 9 | -47.19 | 114.87 | 4.63 | 0.010 |
| -0.059 |  |  | -0.867 |  | -0.185 | -0.316 | -0.323 | -1.058 | 9 | -47.19 | 114.87 | 4.64 | 0.010 |
| -0.064 |  |  | -0.906 | 0.173 |  | -0.357 | -0.399 | -1.038 | 9 | -47.22 | 114.95 | 4.71 | 0.010 |
| -0.239 | + | 0.326 | -0.611 |  |  | -0.526 |  | -1.195 | 9 | -47.22 | 114.95 | 4.71 | 0.010 |
| -0.070 |  | -0.063 | -0.582 | 0.085 |  |  |  | -1.042 | 8 | -48.49 | 114.96 | 4.72 | 0.010 |
| -0.084 |  | 0.202 |  |  | -0.390 |  |  | -0.892 | 7 | -49.74 | 114.99 | 4.75 | 0.010 |
| -0.059 |  |  | -0.537 | 0.190 | -0.256 | -0.319 |  | -1.057 | 9 | -47.27 | 115.04 | 4.80 | 0.010 |
| -0.077 |  |  |  |  |  | -0.154 |  | -0.848 | 6 | -50.98 | 115.09 | 4.85 | 0.009 |
| -0.083 |  | 0.151 |  |  |  |  |  | -0.880 | 6 | -51.02 | 115.17 | 4.93 | 0.009 |
| -0.073 |  | 0.343 | -0.608 | 0.115 |  | -0.543 |  | -1.193 | 9 | -47.34 | 115.19 | 4.95 | 0.009 |
| -0.206 | + |  | -0.608 |  | -0.181 | -0.311 |  | -1.100 | 9 | -47.35 | 115.20 | 4.96 | 0.009 |
| -0.085 |  |  |  |  | -0.366 | -0.146 |  | -0.856 | 7 | -49.87 | 115.24 | 5.00 | 0.009 |
| -0.226 | + |  |  |  | -0.339 |  |  | -0.864 | 7 | -49.88 | 115.27 | 5.03 | 0.009 |
| -0.093 |  | 0.554 |  |  |  | -0.530 |  | -1.004 | 7 | -49.88 | 115.27 | 5.03 | 0.009 |
| -0.081 |  | 0.631 | -0.912 |  | -0.254 | -0.711 | -0.573 | -1.146 | 10 | -46.10 | 115.30 | 5.06 | 0.008 |
| -0.229 | + |  | -0.616 | 0.109 |  | -0.341 |  | -1.101 | 9 | -47.49 | 115.49 | 5.25 | 0.008 |
| -0.081 |  |  |  | 0.379 | -0.414 | -0.243 |  | -0.860 | 8 | -48.78 | 115.53 | 5.29 | 0.007 |
| -0.077 |  |  |  | 0.340 |  | -0.243 |  | -0.869 | 7 | -50.13 | 115.78 | 5.54 | 0.007 |
| -0.080 |  | 0.554 | -0.987 | 0.160 |  | -0.713 | -0.626 | -1.131 | 10 | -46.36 | 115.81 | 5.57 | 0.006 |
| -0.232 | + | 0.531 | -0.982 |  |  | -0.689 | -0.572 | -1.160 | 10 | -46.36 | 115.83 | 5.59 | 0.006 |
| -0.089 |  | 0.552 |  | 0.321 | -0.464 | -0.588 |  | -1.005 | 9 | -47.72 | 115.94 | 5.70 | 0.006 |
| -0.269 | + |  |  |  |  |  | 0.288 | -0.975 | 7 | -50.24 | 115.99 | 5.75 | 0.006 |
| -0.075 |  |  |  |  |  | -0.188 | 0.329 | -0.985 | 7 | -50.25 | 116.02 | 5.78 | 0.006 |
| -0.069 |  |  | -0.667 | 0.160 | -0.246 |  | -0.294 | -0.977 | 9 | -47.76 | 116.02 | 5.78 | 0.006 |
| -0.077 |  |  |  | 0.201 |  |  | 0.234 | -0.948 | 7 | -50.27 | 116.04 | 5.80 | 0.006 |
| -0.076 |  |  |  | 0.262 | -0.383 |  | 0.151 | -0.910 | 8 | -49.10 | 116.18 | 5.94 | 0.005 |
| -0.172 | + |  | -0.708 |  | -0.195 |  | -0.267 | -1.015 | 9 | -47.85 | 116.21 | 5.97 | 0.005 |
| -0.250 | + |  |  | 0.244 |  |  |  | -0.868 | 7 | -50.35 | 116.22 | 5.98 | 0.005 |

**Full model set for Table 1b:** Day 12 nestling survival to the start of the following season.

**Effect sizes are given for centered and scaled continuous variables. Presence of factors in the model is indicated with +.**

| Intercept | Sex | Age | Age next season | Body mass | Group size | Post- lay rain | Pre- lay rain | RTL | df | logLik | AICc | Delta AICc | weight |
| --- | --- | --- | --- | --- | --- | --- | --- | --- | --- | --- | --- | --- | --- |
| 0.604 |  |  | -2.164 | 0.695 |  | -0.596 | -1.703 |  | 8 | -84.68 | 186.41 | 0.00 | 0.084 |
| 1.079 | + |  | -2.468 | 0.849 |  | -0.668 | -1.975 |  | 9 | -83.60 | 186.53 | 0.12 | 0.079 |
| 0.982 | + |  | -2.125 | 0.977 |  |  | -1.896 |  | 8 | -85.00 | 187.05 | 0.64 | 0.061 |
| 0.597 |  |  | -1.733 | 0.642 |  |  | -1.399 |  | 7 | -86.14 | 187.10 | 0.69 | 0.059 |
| 0.435 |  |  | -2.053 | 0.716 | 0.504 |  | -1.600 |  | 8 | -85.06 | 187.16 | 0.76 | 0.057 |
| 0.919 | + |  | -2.398 | 0.895 | 0.532 |  | -1.908 |  | 9 | -83.95 | 187.22 | 0.82 | 0.056 |
| 0.416 |  |  | -2.289 | 0.691 | 0.434 | -0.564 | -1.768 |  | 9 | -84.05 | 187.42 | 1.01 | 0.051 |
| 0.851 | + |  | -2.614 | 0.850 | 0.424 | -0.617 | -2.057 |  | 10 | -83.12 | 187.87 | 1.47 | 0.040 |
| 0.592 |  |  | -2.157 | 0.684 |  | -0.612 | -1.700 | -0.173 | 9 | -84.42 | 188.17 | 1.76 | 0.035 |
| 1.064 | + |  | -2.446 | 0.831 |  | -0.683 | -1.961 | -0.198 | 10 | -83.31 | 188.25 | 1.85 | 0.033 |
| 0.613 |  | 0.024 | -2.171 | 0.700 |  | -0.599 | -1.701 |  | 9 | -84.67 | 188.67 | 2.26 | 0.027 |
| 0.918 | + |  | -1.943 | 0.900 |  |  | -1.707 | -0.195 | 9 | -84.72 | 188.77 | 2.36 | 0.026 |
| 1.099 | + | 0.032 | -2.486 | 0.859 |  | -0.674 | -1.978 |  | 10 | -83.59 | 188.82 | 2.41 | 0.025 |
| 0.569 |  |  | -1.716 | 0.637 |  |  | -1.394 | -0.145 | 8 | -85.97 | 189.00 | 2.59 | 0.023 |
| 0.431 |  |  | -2.050 | 0.702 | 0.480 |  | -1.592 | -0.136 | 9 | -84.91 | 189.15 | 2.74 | 0.021 |
| 0.906 | + |  | -2.371 | 0.870 | 0.498 |  | -1.881 | -0.152 | 10 | -83.81 | 189.24 | 2.83 | 0.020 |
| 0.942 | + | 0.042 | -1.952 | 0.947 |  |  | -1.726 |  | 9 | -84.97 | 189.26 | 2.85 | 0.020 |
| 0.611 |  | 0.035 | -1.747 | 0.648 |  |  | -1.398 |  | 8 | -86.14 | 189.32 | 2.91 | 0.020 |
| 0.419 |  |  | -2.276 | 0.680 | 0.409 | -0.580 | -1.760 | -0.154 | 10 | -83.85 | 189.33 | 2.92 | 0.020 |
| 0.438 |  | 0.068 | -2.086 | 0.733 | 0.520 |  | -1.611 |  | 9 | -85.02 | 189.37 | 2.96 | 0.019 |
| 0.945 | + | 0.088 | -2.480 | 0.932 | 0.562 |  | -1.952 |  | 10 | -83.91 | 189.45 | 3.04 | 0.018 |
| 0.419 |  | 0.014 | -2.294 | 0.694 | 0.436 | -0.565 | -1.769 |  | 10 | -84.05 | 189.72 | 3.32 | 0.016 |
| 0.854 | + |  | -2.578 | 0.829 | 0.387 | -0.636 | -2.031 | -0.176 | 11 | -82.90 | 189.77 | 3.36 | 0.016 |
| 0.783 |  |  | -2.141 |  |  |  | -1.419 |  | 6 | -88.63 | 189.86 | 3.45 | 0.015 |
| 0.749 |  |  | -2.475 |  |  | -0.466 | -1.646 |  | 7 | -87.69 | 190.19 | 3.79 | 0.013 |
| 0.861 | + | 0.028 | -2.636 | 0.861 | 0.430 | -0.621 | -2.065 |  | 11 | -83.12 | 190.21 | 3.80 | 0.013 |
| 0.595 |  | 0.007 | -2.159 | 0.686 |  | -0.612 | -1.700 | -0.172 | 10 | -84.42 | 190.47 | 4.06 | 0.011 |
| 0.464 |  |  | -2.247 |  | 0.513 |  | -1.527 |  | 7 | -87.85 | 190.52 | 4.11 | 0.011 |
| 1.070 | + | 0.010 | -2.452 | 0.834 |  | -0.685 | -1.962 | -0.198 | 11 | -83.31 | 190.59 | 4.18 | 0.010 |
| 0.485 |  |  | -2.561 |  | 0.469 | -0.455 | -1.718 |  | 8 | -87.00 | 191.05 | 4.64 | 0.008 |
| 0.924 | + | 0.025 | -1.954 | 0.909 |  |  | -1.711 | -0.195 | 10 | -84.72 | 191.07 | 4.66 | 0.008 |
| 1.103 | + |  | -2.344 |  |  |  | -1.559 |  | 7 | -88.16 | 191.14 | 4.73 | 0.008 |
| 0.459 |  |  |  | 0.779 |  |  |  |  | 5 | -90.36 | 191.15 | 4.75 | 0.008 |
| 0.578 |  | 0.021 | -1.724 | 0.641 |  |  | -1.394 | -0.143 | 9 | -85.97 | 191.26 | 4.85 | 0.007 |
| 0.434 |  | 0.057 | -2.077 | 0.717 | 0.493 |  | -1.600 | -0.133 | 10 | -84.89 | 191.41 | 5.01 | 0.007 |
| 1.082 | + |  | -2.706 |  |  | -0.503 | -1.806 |  | 8 | -87.19 | 191.43 | 5.02 | 0.007 |
| 0.927 | + | 0.072 | -2.435 | 0.900 | 0.523 |  | -1.916 | -0.149 | 11 | -83.78 | 191.52 | 5.12 | 0.006 |
| 0.418 |  | -0.002 | -2.276 | 0.680 | 0.409 | -0.580 | -1.760 | -0.154 | 11 | -83.85 | 191.67 | 5.26 | 0.006 |
| 0.760 |  |  | -2.117 |  |  |  | -1.414 | -0.146 | 7 | -88.45 | 191.70 | 5.30 | 0.006 |
| 0.727 |  |  | -2.464 |  |  | -0.489 | -1.651 | -0.177 | 8 | -87.42 | 191.89 | 5.48 | 0.005 |
| 0.547 |  |  | -0.434 | 0.663 |  |  |  |  | 6 | -89.68 | 191.96 | 5.55 | 0.005 |
| 0.726 | + |  | -2.454 |  | 0.528 |  | -1.679 |  | 8 | -87.50 | 192.04 | 5.63 | 0.005 |
| 0.791 |  | 0.019 | -2.151 |  |  |  | -1.420 |  | 7 | -88.63 | 192.06 | 5.65 | 0.005 |
| 0.856 | + | 0.007 | -2.583 | 0.832 | 0.388 | -0.637 | -2.033 | -0.176 | 12 | -82.90 | 192.14 | 5.74 | 0.005 |
| 0.774 | + |  |  | 0.887 |  |  |  |  | 6 | -89.84 | 192.27 | 5.87 | 0.004 |

**Full model set for Table 2a:** Day 4 nestling survival to fledging.

**Effect sizes are given for centered and scaled continuous variables. Presence of factors in the model is indicated with +.**

| Intercept | Sex | Age | Body mass | Group size | Post- lay rain | Pre- lay rain | RTL | df | logLik | AICc | Delta AICc | weight |
| --- | --- | --- | --- | --- | --- | --- | --- | --- | --- | --- | --- | --- |
| 0.354 |  |  | 0.578 | -0.424 |  |  | -0.825 | 7 | -47.30 | 110.11 | 0.00 | 0.073 |
| 0.357 |  |  | 0.699 | -0.436 | -0.409 |  | -0.850 | 8 | -46.09 | 110.15 | 0.04 | 0.072 |
| 0.381 |  |  | 0.686 |  | -0.431 |  | -0.888 | 7 | -47.42 | 110.35 | 0.24 | 0.065 |
| 0.367 |  |  | 0.560 |  |  |  | -0.862 | 6 | -48.66 | 110.44 | 0.33 | 0.062 |
| -0.023 | + |  | 0.609 |  | -0.456 |  | -0.912 | 8 | -46.39 | 110.75 | 0.64 | 0.053 |
| 0.027 | + |  | 0.501 |  |  |  | -0.891 | 7 | -47.87 | 111.26 | 1.15 | 0.041 |
| 0.057 | + |  | 0.652 | -0.363 | -0.441 |  | -0.889 | 9 | -45.45 | 111.40 | 1.30 | 0.038 |
| 0.104 | + |  | 0.539 | -0.371 |  |  | -0.858 | 8 | -46.82 | 111.61 | 1.50 | 0.034 |
| 0.352 |  |  | 0.660 | -0.458 |  | -0.223 | -0.752 | 8 | -46.99 | 111.96 | 1.85 | 0.029 |
| 0.352 |  | -0.181 | 0.630 | -0.410 |  |  | -0.797 | 8 | -47.08 | 112.13 | 2.02 | 0.027 |
| 0.353 |  |  | 0.774 | -0.461 | -0.402 | -0.212 | -0.778 | 9 | -45.82 | 112.15 | 2.04 | 0.026 |
| 0.366 |  | -0.225 | 0.620 |  |  |  | -0.823 | 7 | -48.34 | 112.20 | 2.09 | 0.026 |
| -0.055 | + |  |  |  |  |  | -0.845 | 6 | -49.62 | 112.35 | 2.24 | 0.024 |
| 0.329 |  |  |  |  |  |  | -0.804 | 5 | -50.81 | 112.41 | 2.30 | 0.023 |
| 0.361 |  | 0.174 | 0.680 | -0.449 | -0.518 |  | -0.886 | 9 | -45.98 | 112.46 | 2.35 | 0.023 |
| 0.376 |  |  | 0.727 |  | -0.427 | -0.133 | -0.843 | 8 | -47.33 | 112.62 | 2.52 | 0.021 |
| 0.364 |  |  | 0.601 |  |  | -0.129 | -0.820 | 7 | -48.57 | 112.65 | 2.54 | 0.021 |
| 0.384 |  | 0.126 | 0.674 |  | -0.513 |  | -0.913 | 8 | -47.37 | 112.71 | 2.60 | 0.020 |
| 0.322 |  |  |  | -0.343 |  |  | -0.789 | 6 | -49.81 | 112.73 | 2.62 | 0.020 |
| -0.001 | + | -0.264 | 0.564 |  |  |  | -0.838 | 8 | -47.41 | 112.80 | 2.69 | 0.019 |
| -0.034 | + |  | 0.659 |  | -0.455 | -0.152 | -0.859 | 9 | -46.25 | 113.01 | 2.90 | 0.017 |
| -0.107 | + |  |  |  | -0.311 |  | -0.862 | 7 | -48.82 | 113.15 | 3.04 | 0.016 |
| -0.020 | + | 0.077 | 0.601 |  | -0.505 |  | -0.929 | 9 | -46.37 | 113.23 | 3.12 | 0.015 |
| -0.002 | + |  |  | -0.291 |  |  | -0.834 | 7 | -48.91 | 113.33 | 3.22 | 0.015 |
| 0.050 | + |  | 0.735 | -0.391 | -0.439 | -0.220 | -0.819 | 10 | -45.18 | 113.45 | 3.35 | 0.014 |
| 0.021 | + |  | 0.548 |  |  | -0.139 | -0.846 | 8 | -47.76 | 113.49 | 3.38 | 0.013 |
| 0.103 | + |  | 0.626 | -0.407 |  | -0.226 | -0.788 | 9 | -46.52 | 113.53 | 3.42 | 0.013 |
| 0.082 | + | -0.213 | 0.599 | -0.346 |  |  | -0.828 | 9 | -46.52 | 113.55 | 3.44 | 0.013 |
| 0.332 |  |  |  |  | -0.241 |  | -0.813 | 6 | -50.32 | 113.76 | 3.65 | 0.012 |
| 0.066 | + | 0.138 | 0.634 | -0.375 | -0.524 |  | -0.914 | 10 | -45.38 | 113.86 | 3.75 | 0.011 |
| 0.354 |  | 0.373 | 0.771 | -0.509 | -0.628 | -0.352 | -0.795 | 10 | -45.42 | 113.94 | 3.83 | 0.011 |
| 0.324 |  |  |  | -0.336 | -0.222 |  | -0.800 | 7 | -49.38 | 114.28 | 4.17 | 0.009 |
| 0.352 |  | -0.117 | 0.678 | -0.441 |  | -0.177 | -0.751 | 9 | -46.92 | 114.33 | 4.22 | 0.009 |
| -0.055 | + |  |  | -0.269 | -0.291 |  | -0.853 | 8 | -48.24 | 114.45 | 4.34 | 0.008 |
| -0.070 | + | -0.101 |  |  |  |  | -0.826 | 7 | -49.53 | 114.58 | 4.47 | 0.008 |
| 0.364 |  | -0.207 | 0.634 |  |  | -0.059 | -0.807 | 8 | -48.33 | 114.62 | 4.52 | 0.008 |
| 0.333 |  |  |  |  |  | 0.083 | -0.833 | 6 | -50.76 | 114.64 | 4.53 | 0.008 |
| -0.051 | + |  |  |  |  | 0.054 | -0.864 | 7 | -49.59 | 114.70 | 4.59 | 0.007 |
| 0.328 |  | -0.038 |  |  |  |  | -0.797 | 6 | -50.80 | 114.72 | 4.61 | 0.007 |
| 0.378 |  | 0.236 | 0.727 |  | -0.577 | -0.212 | -0.861 | 9 | -47.17 | 114.85 | 4.74 | 0.007 |
| 0.323 |  |  |  | -0.340 |  | 0.029 | -0.798 | 7 | -49.80 | 115.11 | 5.00 | 0.006 |
| 0.322 |  | 0.004 |  | -0.344 |  |  | -0.789 | 7 | -49.81 | 115.12 | 5.02 | 0.006 |
| -0.097 | + | 0.204 |  |  | -0.445 |  | -0.899 | 8 | -48.64 | 115.25 | 5.14 | 0.006 |
| -0.002 | + | -0.247 | 0.579 |  |  | -0.057 | -0.823 | 9 | -47.40 | 115.29 | 5.18 | 0.005 |
| -0.028 | + | 0.186 | 0.660 |  | -0.575 | -0.215 | -0.877 | 10 | -46.15 | 115.40 | 5.29 | 0.005 |
| 0.068 | + | 0.326 | 0.733 | -0.436 | -0.635 | -0.338 | -0.835 | 11 | -44.87 | 115.50 | 5.39 | 0.005 |
| -0.100 | + |  |  |  | -0.316 | 0.079 | -0.888 | 8 | -48.77 | 115.52 | 5.41 | 0.005 |
| 0.340 |  | 0.267 |  |  | -0.425 |  | -0.870 | 7 | -50.04 | 115.60 | 5.49 | 0.005 |
| -0.010 | + | -0.051 |  | -0.283 |  |  | -0.827 | 8 | -48.89 | 115.75 | 5.64 | 0.004 |
| -0.001 | + |  |  | -0.290 |  | 0.010 | -0.837 | 8 | -48.91 | 115.79 | 5.68 | 0.004 |
| 0.086 | + | -0.154 | 0.647 | -0.378 |  | -0.167 | -0.786 | 10 | -46.38 | 115.87 | 5.76 | 0.004 |
| 0.327 |  | 0.318 |  | -0.365 | -0.433 |  | -0.859 | 8 | -48.97 | 115.92 | 5.81 | 0.004 |
| 0.338 |  |  |  |  | -0.252 | 0.110 | -0.853 | 7 | -50.24 | 115.98 | 5.88 | 0.004 |

**Full model set for Table 2b:** Day 4 nestling survival to fledging, excluding ‘predated’ nestlings.

**Effect sizes are given for centered and scaled continuous variables. Presence of factors in the model is indicated with +.**

| Intercept | Sex | Age | Body mass | Group size | Post- lay rain | Pre- lay rain | RTL | df | logLik | AICc | Delta AICc | weight |
| --- | --- | --- | --- | --- | --- | --- | --- | --- | --- | --- | --- | --- |
| 0.859 |  |  | 0.571 |  |  |  |  | 5 | -39.86 | 90.69 | 0.00 | 0.122 |
| 0.886 |  |  | 0.580 |  |  |  | -0.328 | 6 | -39.17 | 91.72 | 1.03 | 0.073 |
| 0.642 | + |  | 0.542 |  |  |  |  | 6 | -39.56 | 92.49 | 1.80 | 0.049 |
| 0.864 |  |  | 0.613 |  | -0.195 |  |  | 6 | -39.60 | 92.58 | 1.89 | 0.047 |
| 0.806 |  |  |  |  |  |  |  | 4 | -42.03 | 92.70 | 2.01 | 0.044 |
| 0.859 |  | -0.066 | 0.590 |  |  |  |  | 6 | -39.83 | 93.05 | 2.36 | 0.037 |
| 0.859 |  |  | 0.577 |  |  | -0.016 |  | 6 | -39.86 | 93.10 | 2.41 | 0.037 |
| 0.859 |  |  | 0.572 | -0.010 |  |  |  | 6 | -39.86 | 93.10 | 2.41 | 0.036 |
| 0.616 | + |  | 0.543 |  |  |  | -0.376 | 7 | -38.71 | 93.28 | 2.59 | 0.033 |
| 0.896 |  |  | 0.630 |  | -0.227 |  | -0.350 | 7 | -38.83 | 93.54 | 2.85 | 0.029 |
| 0.823 |  |  |  |  |  |  | -0.310 | 5 | -41.35 | 93.67 | 2.98 | 0.027 |
| 0.511 | + |  |  |  |  |  |  | 5 | -41.41 | 93.80 | 3.11 | 0.026 |
| 0.888 |  |  | 0.557 |  |  | 0.076 | -0.348 | 7 | -39.14 | 94.15 | 3.46 | 0.022 |
| 0.887 |  |  | 0.585 | -0.052 |  |  | -0.335 | 7 | -39.15 | 94.18 | 3.49 | 0.021 |
| 0.886 |  | -0.031 | 0.589 |  |  |  | -0.324 | 7 | -39.17 | 94.20 | 3.51 | 0.021 |
| 0.625 | + |  | 0.580 |  | -0.219 |  |  | 7 | -39.24 | 94.36 | 3.67 | 0.019 |
| 0.478 | + |  |  |  |  |  | -0.370 | 6 | -40.52 | 94.41 | 3.72 | 0.019 |
| 0.811 |  |  |  |  |  | 0.165 |  | 5 | -41.85 | 94.67 | 3.98 | 0.017 |
| 0.624 | + | -0.109 | 0.569 |  |  |  |  | 7 | -39.49 | 94.84 | 4.15 | 0.015 |
| 0.808 |  | 0.101 |  |  |  |  |  | 5 | -41.96 | 94.89 | 4.20 | 0.015 |
| 0.624 | + |  | 0.534 | 0.059 |  |  |  | 7 | -39.54 | 94.94 | 4.25 | 0.015 |
| 0.632 | + |  | 0.561 |  |  | -0.060 |  | 7 | -39.54 | 94.94 | 4.25 | 0.015 |
| 0.807 |  |  |  |  | -0.072 |  |  | 5 | -42.00 | 94.96 | 4.27 | 0.014 |
| 0.866 |  | 0.115 | 0.596 |  | -0.266 |  |  | 7 | -39.56 | 94.98 | 4.29 | 0.014 |
| 0.596 | + |  | 0.590 |  | -0.260 |  | -0.405 | 8 | -38.29 | 95.01 | 4.32 | 0.014 |
| 0.806 |  |  |  | 0.036 |  |  |  | 5 | -42.03 | 95.02 | 4.33 | 0.014 |
| 0.865 |  |  | 0.622 |  | -0.197 | -0.025 |  | 7 | -39.60 | 95.07 | 4.38 | 0.014 |
| 0.864 |  |  | 0.615 | -0.022 | -0.197 |  |  | 7 | -39.60 | 95.07 | 4.38 | 0.014 |
| 0.839 |  |  |  |  |  | 0.260 | -0.376 | 6 | -40.96 | 95.29 | 4.60 | 0.012 |
| 0.859 |  | -0.066 | 0.591 | -0.011 |  |  |  | 7 | -39.83 | 95.53 | 4.84 | 0.011 |
| 0.859 |  | -0.069 | 0.588 |  |  | 0.007 |  | 7 | -39.83 | 95.54 | 4.85 | 0.011 |
| 0.859 |  |  | 0.580 | -0.017 |  | -0.022 |  | 7 | -39.86 | 95.58 | 4.89 | 0.011 |
| 0.903 |  | 0.230 | 0.598 |  | -0.373 |  | -0.391 | 8 | -38.67 | 95.77 | 5.08 | 0.010 |
| 0.604 | + | -0.077 | 0.563 |  |  |  | -0.369 | 8 | -38.67 | 95.79 | 5.10 | 0.010 |
| 0.827 |  | 0.136 |  |  |  |  | -0.326 | 6 | -41.23 | 95.83 | 5.14 | 0.009 |
| 0.621 | + |  | 0.534 |  |  | 0.032 | -0.384 | 8 | -38.70 | 95.84 | 5.15 | 0.009 |
| 0.610 | + |  | 0.541 | 0.021 |  |  | -0.373 | 8 | -38.70 | 95.85 | 5.16 | 0.009 |
| 0.825 |  |  |  |  | -0.100 |  | -0.319 | 6 | -41.28 | 95.94 | 5.25 | 0.009 |
| 0.494 | + |  |  |  | -0.120 |  |  | 6 | -41.31 | 96.01 | 5.32 | 0.009 |
| 0.479 | + |  |  | 0.124 |  |  |  | 6 | -41.31 | 96.01 | 5.32 | 0.009 |
| 0.897 |  |  | 0.637 | -0.071 | -0.234 |  | -0.361 | 8 | -38.80 | 96.05 | 5.36 | 0.008 |
| 0.898 |  |  | 0.606 |  | -0.226 | 0.076 | -0.371 | 8 | -38.80 | 96.05 | 5.36 | 0.008 |
| 0.536 | + |  |  |  |  | 0.108 |  | 6 | -41.34 | 96.06 | 5.37 | 0.008 |
| 0.823 |  |  |  | 0.001 |  |  | -0.310 | 6 | -41.35 | 96.08 | 5.39 | 0.008 |
| 0.520 | + | 0.039 |  |  |  |  |  | 6 | -41.40 | 96.19 | 5.50 | 0.008 |
| 0.523 | + |  |  |  |  | 0.203 | -0.417 | 7 | -40.30 | 96.46 | 5.77 | 0.007 |
| 0.455 | + |  |  |  | -0.158 |  | -0.387 | 7 | -40.35 | 96.57 | 5.88 | 0.006 |
| 0.888 |  | -0.060 | 0.567 |  |  | 0.097 | -0.346 | 8 | -39.12 | 96.69 | 6.00 | 0.006 |

**Full model set for Table 2c:** Day 4 nestling survival to fledging, excluding ‘expired nestlings.

Effect sizes are given for centered and scaled continuous variables. Presence of factors in the model is indicated with +.

| Intercept | Sex | Age | Body mass | Group size | Post- lay rain | Pre- lay rain | RTL | df | logLik | AICc | Delta AICc | weight |
| --- | --- | --- | --- | --- | --- | --- | --- | --- | --- | --- | --- | --- |
| 0.941 |  |  |  |  | -0.673 |  | -1.229 | 6 | -40.21 | 93.67 | 0.00 | 0.071 |
| 0.825 |  |  |  | -0.547 | -0.571 |  | -1.102 | 7 | -38.99 | 93.68 | 0.00 | 0.071 |
| 8.242 |  |  |  |  |  |  |  | 4 | -42.59 | 93.75 | 0.08 | 0.068 |
| 0.815 |  |  |  | -0.565 |  |  | -1.037 | 6 | -40.50 | 94.25 | 0.58 | 0.053 |
| 0.899 |  |  |  |  |  |  | -1.150 | 5 | -41.92 | 94.72 | 1.05 | 0.042 |
| 0.552 | + |  |  |  | -0.649 |  | -1.185 | 7 | -39.87 | 95.43 | 1.76 | 0.029 |
| 9.214 | + |  |  |  |  |  |  | 5 | -42.31 | 95.50 | 1.83 | 0.028 |
| 0.829 |  |  | 0.266 | -0.587 | -0.617 |  | -1.080 | 8 | -38.66 | 95.53 | 1.86 | 0.028 |
| 7.897 |  |  |  |  | -0.786 |  |  | 5 | -42.38 | 95.64 | 1.96 | 0.027 |
| 0.957 |  |  | 0.219 |  | -0.720 |  | -1.225 | 7 | -40.02 | 95.74 | 2.06 | 0.025 |
| 0.564 | + |  |  | -0.489 | -0.561 |  | -1.069 | 8 | -38.81 | 95.83 | 2.16 | 0.024 |
| 0.812 |  | -0.293 |  | -0.541 |  |  | -1.006 | 7 | -40.09 | 95.89 | 2.21 | 0.023 |
| 8.327 |  |  | -0.337 |  |  |  |  | 5 | -42.51 | 95.90 | 2.23 | 0.023 |
| 8.234 |  |  |  |  |  | -0.170 |  | 5 | -42.57 | 96.03 | 2.35 | 0.022 |
| 0.942 |  | 0.131 |  |  | -0.763 |  | -1.252 | 7 | -40.17 | 96.04 | 2.37 | 0.022 |
| 0.888 |  | -0.353 |  |  |  |  | -1.100 | 6 | -41.40 | 96.05 | 2.37 | 0.022 |
| 0.822 |  | 0.149 |  | -0.551 | -0.669 |  | -1.125 | 8 | -38.93 | 96.08 | 2.41 | 0.021 |
| 0.938 |  |  |  |  | -0.671 | -0.022 | -1.221 | 7 | -40.21 | 96.11 | 2.44 | 0.021 |
| 0.816 |  |  |  | -0.548 | -0.561 | -0.072 | -1.075 | 8 | -38.97 | 96.15 | 2.48 | 0.021 |
| 0.813 |  |  | 0.170 | -0.588 |  |  | -1.018 | 7 | -40.36 | 96.41 | 2.74 | 0.018 |
| 0.651 | + |  |  | -0.533 |  |  | -1.029 | 7 | -40.41 | 96.52 | 2.84 | 0.017 |
| 0.804 |  |  |  | -0.571 |  | -0.120 | -0.998 | 7 | -40.44 | 96.58 | 2.90 | 0.017 |
| 0.623 | + |  |  |  |  |  | -1.135 | 6 | -41.70 | 96.66 | 2.99 | 0.016 |
| 8.383 |  | 1.428 |  |  | -1.952 |  |  | 6 | -41.73 | 96.71 | 3.04 | 0.016 |
| 0.903 |  |  | 0.101 |  |  |  | -1.145 | 6 | -41.88 | 97.01 | 3.33 | 0.013 |
| 0.895 |  |  |  |  |  | -0.059 | -1.132 | 6 | -41.91 | 97.07 | 3.39 | 0.013 |
| 5.547 |  |  |  | -1.618 | -3.187 |  |  | 6 | -42.03 | 97.31 | 3.63 | 0.012 |
| 0.584 | + |  | 0.178 |  | -0.684 |  | -1.180 | 8 | -39.73 | 97.67 | 4.00 | 0.010 |
| 9.156 | + |  | -0.184 |  |  |  |  | 6 | -42.29 | 97.83 | 4.16 | 0.009 |
| 0.563 | + | -0.368 |  |  |  |  | -1.073 | 7 | -41.09 | 97.88 | 4.21 | 0.009 |
| 0.811 |  | -0.340 | 0.232 | -0.570 |  |  | -0.975 | 8 | -39.83 | 97.88 | 4.21 | 0.009 |
| 0.614 | + |  | 0.234 | -0.533 | -0.602 |  | -1.055 | 9 | -38.54 | 97.89 | 4.22 | 0.009 |
| 0.559 | + | 0.092 |  |  | -0.712 |  | -1.203 | 8 | -39.85 | 97.91 | 4.24 | 0.009 |
| 0.806 |  |  | 0.303 | -0.594 | -0.599 | -0.161 | -1.012 | 9 | -38.56 | 97.93 | 4.26 | 0.008 |
| 0.545 | + |  |  |  | -0.645 | -0.043 | -1.170 | 8 | -39.86 | 97.93 | 4.26 | 0.008 |
| 0.827 |  | 0.100 | 0.256 | -0.589 | -0.681 |  | -1.096 | 9 | -38.63 | 98.08 | 4.41 | 0.008 |
| 0.601 | + | -0.306 |  | -0.497 |  |  | -0.988 | 8 | -39.95 | 98.12 | 4.45 | 0.008 |
| 0.946 |  |  | 0.237 |  | -0.711 | -0.089 | -1.193 | 8 | -40.00 | 98.21 | 4.53 | 0.007 |
| 0.894 |  | -0.393 | 0.178 |  |  |  | -1.086 | 7 | -41.26 | 98.21 | 4.54 | 0.007 |
| 0.957 |  | 0.091 | 0.210 |  | -0.780 |  | -1.240 | 8 | -40.00 | 98.22 | 4.55 | 0.007 |
| 0.572 | + | 0.118 |  | -0.495 | -0.639 |  | -1.090 | 9 | -38.77 | 98.35 | 4.68 | 0.007 |
| 0.543 | + |  |  | -0.489 | -0.549 | -0.087 | -1.034 | 9 | -38.77 | 98.36 | 4.69 | 0.007 |
| 5.734 | + |  |  |  | -4.780 |  |  | 6 | -42.57 | 98.39 | 4.71 | 0.007 |
| 0.813 |  | -0.300 |  | -0.539 |  | 0.017 | -1.011 | 8 | -40.09 | 98.40 | 4.73 | 0.007 |
| 0.896 |  | -0.398 |  |  |  | 0.112 | -1.128 | 7 | -41.36 | 98.41 | 4.74 | 0.007 |
| 7.948 |  | 1.467 |  | -1.241 | -2.411 |  |  | 7 | -41.37 | 98.43 | 4.76 | 0.007 |
| 9.821 | + | 1.663 |  |  | -1.864 |  |  | 7 | -41.39 | 98.47 | 4.80 | 0.006 |
| 0.794 |  | 0.256 |  | -0.558 | -0.713 | -0.176 | -1.073 | 9 | -38.84 | 98.49 | 4.82 | 0.006 |
| 0.931 |  | 0.187 |  |  | -0.790 | -0.095 | -1.229 | 8 | -40.15 | 98.52 | 4.85 | 0.006 |
| 9.309 |  | 2.024 | -0.826 |  | -2.064 |  |  | 7 | -41.46 | 98.62 | 4.95 | 0.006 |
| 5.515 |  |  |  |  | -4.243 | 0.330 |  | 6 | -42.70 | 98.65 | 4.98 | 0.006 |
| 0.795 |  |  | 0.216 | -0.603 |  | -0.184 | -0.950 | 8 | -40.22 | 98.66 | 4.99 | 0.006 |
| 5.402 |  |  | -0.184 |  | -4.321 |  |  | 6 | -42.72 | 98.69 | 5.01 | 0.006 |
| 7.664 |  | 3.010 |  | -4.752 | -5.030 | -3.906 |  | 8 | -40.26 | 98.73 | 5.06 | 0.006 |
| 0.673 | + |  | 0.154 | -0.557 |  |  | -1.012 | 8 | -40.29 | 98.80 | 5.13 | 0.005 |
| 0.634 | + |  |  | -0.539 |  | -0.125 | -0.987 | 8 | -40.34 | 98.90 | 5.23 | 0.005 |
| 8.293 |  | 1.515 |  |  | -1.894 | -0.617 |  | 7 | -41.61 | 98.92 | 5.25 | 0.005 |
| 12.036 | + | 5.379 |  | -7.531 | -7.333 | -5.591 |  | 9 | -39.09 | 98.99 | 5.32 | 0.005 |
| 0.633 | + |  | 0.078 |  |  |  | -1.131 | 7 | -41.68 | 99.05 | 5.38 | 0.005 |
| 0.616 | + |  |  |  |  | -0.070 | -1.114 | 7 | -41.68 | 99.07 | 5.39 | 0.005 |
| 6.251 | + |  |  | -1.882 | -3.696 |  |  | 7 | -41.77 | 99.24 | 5.56 | 0.004 |
| 0.896 |  |  | 0.122 |  |  | -0.093 | -1.116 | 7 | -41.85 | 99.39 | 5.72 | 0.004 |
| 7.162 |  |  |  | -3.990 | -2.857 | -1.617 |  | 7 | -41.91 | 99.52 | 5.85 | 0.004 |

**Full model set for Table 3a:** Survival to the following season of nestlings with RTL measures at day 4 and day 12. Effect sizes are given for centered and scaled continuous variables.

| Intercept | Age next season | Delta RTL | Delta RTL^2^ | Pre- lay rain | D4 RTL | D12 body mass | df | logLik | AICc | Delta AICc | weight |
| --- | --- | --- | --- | --- | --- | --- | --- | --- | --- | --- | --- |
| 1.150 |  | 1.036 | -1.180 |  |  | 1.007 | 6 | -18.34 | 51.30 | 0.00 | 0.213 |
| 1.366 |  | 0.747 | -1.351 |  |  |  | 5 | -20.26 | 52.33 | 1.03 | 0.127 |
| 1.430 |  | 0.984 | -1.449 | 0.718 |  |  | 6 | -19.24 | 53.10 | 1.80 | 0.086 |
| 1.416 | -0.655 | 1.056 | -1.486 |  |  |  | 6 | -19.31 | 53.24 | 1.94 | 0.081 |
| 1.177 |  | 1.115 | -1.230 | 0.456 |  | 0.917 | 7 | -17.87 | 53.36 | 2.06 | 0.076 |
| 1.176 | -0.374 | 1.141 | -1.248 |  |  | 0.898 | 7 | -18.03 | 53.68 | 2.38 | 0.065 |
| 0.306 |  | 0.862 |  |  |  | 1.299 | 5 | -21.20 | 54.22 | 2.92 | 0.050 |
| 1.148 |  | 0.986 | -1.172 |  | -0.057 | 0.996 | 7 | -18.33 | 54.28 | 2.98 | 0.048 |
| 1.350 |  | 0.559 | -1.317 |  | -0.238 |  | 6 | -20.14 | 54.90 | 3.60 | 0.035 |
| 1.421 |  | 0.696 | -1.387 | 0.814 | -0.398 |  | 7 | -18.94 | 55.50 | 4.20 | 0.026 |
| 1.399 | -0.729 | 0.773 | -1.424 |  | -0.388 |  | 7 | -19.02 | 55.66 | 4.36 | 0.024 |
| 1.178 | 2.143 | 0.899 | -1.100 | 2.571 |  | 1.125 | 8 | -17.52 | 55.85 | 4.55 | 0.022 |
| 0.381 |  |  |  |  | -0.688 | 0.981 | 5 | -22.10 | 56.02 | 4.72 | 0.020 |
| 0.358 |  |  |  |  |  | 0.903 | 4 | -23.45 | 56.08 | 4.78 | 0.020 |
| 1.436 | 0.265 | 0.951 | -1.432 | 0.996 |  |  | 7 | -19.23 | 56.08 | 4.78 | 0.020 |
| 0.294 |  | 0.835 |  | 0.363 |  | 1.283 | 6 | -20.79 | 56.20 | 4.90 | 0.018 |
| 1.177 |  | 0.957 | -1.207 | 0.504 | -0.188 | 0.870 | 8 | -17.81 | 56.42 | 5.13 | 0.016 |
| 0.291 | -0.242 | 0.851 |  |  |  | 1.276 | 6 | -21.01 | 56.65 | 5.35 | 0.015 |
| 1.174 | -0.423 | 0.990 | -1.227 |  | -0.185 | 0.849 | 8 | -17.97 | 56.75 | 5.45 | 0.014 |
| 0.314 |  | 0.737 |  |  | -0.199 | 1.247 | 6 | -21.11 | 56.85 | 5.55 | 0.013 |
| 0.382 |  |  |  | 0.544 | -0.711 | 0.902 | 6 | -21.29 | 57.21 | 5.91 | 0.011 |

**Full model set for Table 3b:** Survival to the following season of nestlings with RTL measures at day 4 and day 12, where delta RTL is below the peak of the quadratic. Effect sizes are given for centered and scaled continuous variables.

| **Intercept** | **Delta RTL** | **D4 RTL** | **D12 body mass** | **df** | **logLik** | **AICc** | **delta** | **weight** |
| --- | --- | --- | --- | --- | --- | --- | --- | --- |
| **0.533** | 2.033 |  |  | 2 | -11.69 | 27.90 | 0.00 | 0.521 |
| **0.450** | 1.934 |  | 0.757 | 3 | -11.08 | 29.26 | 1.36 | 0.263 |
| **0.547** | 1.900 | -0.191 |  | 3 | -11.64 | 30.37 | 2.47 | 0.151 |
| **0.447** | 1.947 | 0.019 | 0.764 | 4 | -11.08 | 32.07 | 4.17 | 0.065 |

**Full model set for Table 3c: Survival to the following season of nestlings with RTL measures at day 4 and day 12, where delta RTL is above the peak of the quadratic. Effect sizes are given for centered and scaled continuous variables.**

| Intercept | z.dtel.days | z.RTL4 | z.Weight12 | df | logLik | AICc | delta | weight |
| --- | --- | --- | --- | --- | --- | --- | --- | --- |
| 0.154 |  |  |  | 3 | -8.97 | 26.61 | 0 | 0.541 |
| 0.086 |  |  | 1.216 | 4 | -7.43 | 27.87 | 1.25 | 0.289 |
| 0.17**1** |  | -0.586 |  | 4 | -8.50 | 30.00 | 3.38 | 0.099 |
| 0.155 | -0.282 |  |  | 4 | -8.86 | 30.71 | 4.10 | 0.070 |

**S3.3 Effect of outlier removal on whether within-individual rate of change in telomere length predicts survival**

The quadratic relationship between rate of change in telomere length and survival to the following season was robust to the exclusion of a possible outlier in the rate of change in telomere length data, with the linear and quadratic terms both retained within the top 2 models (Table S3.2)

**Table S3.2. Model investigating whether a nestling’s rate of change in telomere length (ΔRTL) from day 4 to day 12 predicts their survival to the start of the following breeding season after removal of an outlier (n=38 birds; see Figure 2). The Δ6 AICc top model set is shown relative to the null model (in grey). Effect sizes are given with standard errors in parentheses. Continuous variables were centred and scaled. Int = intercept, AW = adjusted weight after implementation of the model nesting rule (Richards et al., 2011).**

| Int | Day 12 Body mass | Day 4 RTL | Δ RTL | Δ RTL^2^ | df | logLik | AICc | Δ AICc | AW |
| --- | --- | --- | --- | --- | --- | --- | --- | --- | --- |
| 1.150 | 1.007 (0.573) |  | 1.036 (0.606) | -1.180 (0.631) | 6 | -18.337 | 51.4 | 0.00 | 0.506 |
| 1.366 |  |  | 0.747 (0.516) | -1.351 (0.574) | 5 | -20.256 | 52.4 | 1.00 | 0.306 |
| 0.448 | 0.998 (0.452) |  |  |  | 4 | -22.078 | 53.4 | 1.98 | 0.188 |
| 0.427 |  |  |  |  | 3 | -25.491 | 57.7 | 6.30 | NA |

**S3.4 Models of survival to the following season for individuals showing a) a decrease and b) an increase in RTL between 4 and 12 days of age.**

To confirm our findings that change in telomere length predicts survival of individuals that show an apparent decrease in RTL, but not those with an apparent increase in telomere length, we split the data at 0 (rather than at the peak of the quadratic) and re-ran the models. Top model sets are shown below in Table S2.3.

**Table S3.3. Models investigating whether a nestling’s rate of change in telomere length (ΔRTL) from day 4 to day 12 predicts their survival to the start of the following breeding season, for (a) ΔRTL data *below* 0, and (b) for ΔRTL data *above* 0. The Δ6 AICc top model sets are shown after applying the model nesting rule (Richards, Whittingham, & Stephens, 2011). Effect sizes are given with standard errors in parentheses. Continuous variables were centred and scaled. Int = intercept, Body mass = body mass at day 12, Δ RTL = rate of change in RTL from day 4 to day 12.**

| Int |  | Body mass | Δ RTL | df | logLik | AICc | Δ AICc | AW |
| --- | --- | --- | --- | --- | --- | --- | --- | --- |
| ***(a) data below 0*** *(n = 18)* | | | | | | | | |
| -0.51 (0.75) |  |  | 2.83 (1.32) | 2 | -7.64 | 20.1 | 0 | 1 |
| ***(a) data above 0*** *(n = 21)* | | | | | | | | |
| 0.77 (0.52) |  | 1.03 (0.58) |  | 4 | -11.32 | 33.1 | 0 | 0.623 |
| 0.69 (0.46) |  |  |  | 3 | -13.37 | 34.1 | 1.01 | 0.377 |

References

Angelier, F., Vleck, C. M., Holberton, R. L., & Marra, P. P. (2013). Telomere length, non-breeding habitat and return rate in male American redstarts. *Functional Ecology*, *27*(2), 342–350. doi:10.1111/1365-2435.12041

Asghar, M., Hasselquist, D., Hansson, B., Zehtindjiev, P., Westerdahl, H., & Bensch, S. (2015). Hidden costs of infection: Chronic malaria accelerates telomere degradation and senescence in wild birds. *Science*, *347*(6220), 436–438. doi:10.1126/science.1261121

Barrett, E. L. B., Burke, T. A., Hammers, M., Komdeur, J., & Richardson, D. S. (2013). Telomere length and dynamics predict mortality in a wild longitudinal study. *Molecular Ecology*, *22*(1), 249–259. doi:10.1111/mec.12110

Bize, P., Criscuolo, F., Metcalfe, N. B., Nasir, L., & Monaghan, P. (2009). Telomere dynamics rather than age predict life expectancy in the wild. *Proceedings of the Royal Society B: Biological Sciences*, *276*(1662), 1679–1683. doi:10.1098/rspb.2008.1817

Boonekamp, J. J., Mulder, G. A., Salomons, H. M., Dijkstra, C., & Verhulst, S. (2014). Nestling telomere shortening, but not telomere length, reflects developmental stress and predicts survival in wild birds. *Proceedings of the Royal Society B: Biological Sciences*, *281*(1785), 20133287. doi:10.1098/rspb.2013.3287

Caprioli, M., Romano, M., Romano, A., Rubolini, D., Motta, R., Folini, M., & Saino, N. (2013). Nestling telomere length does not predict longevity, but covaries with adult body size in wild barn swallows. *Biology Letters*, *9*(5), 20130340. doi:10.1098/rsbl.2013.0340

Cerchiara, J. A., Risques, R. A., Prunkard, D., Smith, J. R., Kane, O. J., & Boersma, P. D. (2017). Telomeres shorten and then lengthen before fledging in Magellanic penguins (Spheniscus magellanicus). *Aging*, *9*(2), 489–493. doi:10.18632/aging.101172

Fairlie, J., Holland, R., Pilkington, J. G., Pemberton, J. M., Harrington, L., & Nussey, D. H. (2016). Lifelong leukocyte telomere dynamics and survival in a free-living mammal. *Aging Cell*, *15*(1), 140–148. doi:10.1111/acel.12417

Foote, C. G., Daunt, F., González-Solís, J., Nasir, L., Phillips, R. A., & Monaghan, P. (2011). Individual state and survival prospects: Age, sex, and telomere length in a long-lived seabird. *Behavioral Ecology*, *22*(1), 156–161. doi:10.1093/beheco/arq178

Geiger, S., Le Vaillant, M., Lebard, T., Reichert, S., Stier, A., Le Maho, Y., & Criscuolo, F. (2012). Catching-up but telomere loss: Half-opening the black box of growth and ageing trade-off in wild king penguin chicks. *Molecular Ecology*, *21*(6), 1500–1510. doi:10.1111/j.1365-294X.2011.05331.x

Haussmann, M. F., Winkler, D. W., & Vleck, C. M. (2005). Longer telomeres associated with higher survival in birds. *Biology Letters*, *1*(2), 212–214. doi:10.1098/rsbl.2005.0301

Ouyang, J. Q., Lendvai, Z., Moore, I. T., Bonier, F., & Haussmann, M. F. (2016). Do hormones, telomere lengths, and oxidative stress form an integrated phenotype? A case study in free-living tree swallows. *Integrative and Comparative Biology*, *56*(2), 138–145. doi:10.1093/icb/icw044

Pauliny, A., Wagner, R. H., Augustin, J., Szép, T., & Blomqvist, D. (2006). Age-independent telomere length predicts fitness in two bird species. *Molecular Ecology*, *15*(6), 1681–1687. doi:10.1111/j.1365-294X.2006.02862.x

Pfaffl, M. W. (2001). A new mathematical model for relative quantification in real-time RT-PCR. *Nucleic Acids Research*, *29*(9), 45e–45. doi:10.1093/nar/29.9.e45

Richards, S. A., Whittingham, M. J., & Stephens, P. A. (2011). Model selection and model averaging in behavioural ecology: The utility of the IT-AIC framework. *Behavioral Ecology and Sociobiology*, *65*(1), 77–89. doi:10.1007/s00265-010-1035-8

Salmón, P., Nilsson, J. F., Watson, H., Bensch, S., & Isaksson, C. (2017). Selective disappearance of great tits with short telomeres in urban areas. *Proceedings of the Royal Society B: Biological Sciences*, *284*(1862), 20171349. doi:10.1098/rspb.2017.1349

Salomons, H. M., Mulder, G. A., van de Zande, L., Haussmann, M. F., Linskens, M. H. K., & Verhulst, S. (2009). Telomere shortening and survival in free-living corvids. *Proceedings of the Royal Society B: Biological Sciences*, *276*(1670), 3157–3165. doi:10.1098/rspb.2009.0517

Stier, A., Viblanc, V. A., Massemin-Challet, S., Handrich, Y., Zahn, S., Rojas, E. R., … Criscuolo, F. (2014). Starting with a handicap: Phenotypic differences between early- and late-born king penguin chicks and their survival correlates. *Functional Ecology*, *28*(3), 601–611. doi:10.1111/1365-2435.12204

Taff, C. C., & Freeman-Gallant, C. R. (2017). Sexual signals reflect telomere dynamics in a wild bird. *Ecology and Evolution*, *7*(10), 3436–3442. doi:10.1002/ece3.2948

Ujvari, B., Biro, P. A., Charters, J. E., Brown, G., Heasman, K., Beckmann, C., & Madsen, T. (2017). Curvilinear telomere length dynamics in a squamate reptile. *Functional Ecology*, *31*(3), 753–759. doi:10.1111/1365-2435.12764

Ujvari, B., & Madsen, T. (2009). Short telomeres in hatchling snakes: Erythrocyte telomere dynamics and longevity in tropical pythons. *PLoS ONE*, *4*(10), 2–6. doi:10.1371/journal.pone.0007493

Watson, H., Bolton, M., & Monaghan, P. (2015). Variation in early-life telomere dynamics in a long-lived bird: links to environmental conditions and survival. *Journal of Experimental Biology*, *218*(5), 668–674. doi:10.1242/jeb.104265

Young, R. C., Welcker, J., Barger, C. P., Hatch, S. A., Merkling, T., Kitaiskaia, E. V., … Kitaysky, A. S. (2017). Effects of developmental conditions on growth, stress and telomeres in black-legged kittiwake chicks. *Molecular Ecology*, *26*(13), 3572–3584. doi:10.1111/mec.14121
